# Designing Contact Independent High‐Performance Low‐Cost Flexible Electronics

**DOI:** 10.1002/adma.202410442

**Published:** 2024-10-09

**Authors:** Matthew Waldrip, Yue Yu, Derek Dremann, Tommaso Losi, Benjamin Willner, Mario Caironi, Iain McCulloch, Oana D. Jurchescu

**Affiliations:** ^1^ Department of Physics and Center for Functional Materials Wake Forest University Winston‐Salem NC 27109 USA; ^2^ Center for Nano Science and Technology Istituto Italiano di Tecnologia Via Rubattino 81 Milano 20134 Italy; ^3^ Department of Chemistry Chemistry Research Laboratory University of Oxford Oxford OX1 3TA UK; ^4^ Andlinger Center for Energy and the Environment and Department of Electrical and Computer Engineering Princeton University Princeton NJ 08544 USA

**Keywords:** charge injection, contact resistances, mobilities, organic semiconductors, organic transistors

## Abstract

Organic semiconductors enable low‐cost solution processing of optoelectronic devices on flexible substrates. Their use in contemporary applications, however, is sparse due to persistent challenges in achieving the requisite performance levels in a reliable and reproducible manner. A critical bottleneck is the inefficiency associated with charge injection. Here, large‐scale simulations are employed to identify operational windows where key device parameters that are difficult to control experimentally, such as the contact resistance, become less consequential to overall device functionality. This design methodology overcomes injection barrier limitations in organic field‐effect transistors (OFETs), leading to high charge carrier mobility and significantly expanding the range of suitable electrode materials. Leveraging this new understanding, all‐organic, solution‐deposited OFETs are successfully fabricated on flexible substrates. These devices incorporate printed contacts and showcase mobilities exceeding 5 cm^2^ Vs^−1^. These results provide a route for accessing the fundamental limits of material properties even in the absence of ideal contacts – a critical step in establishing reliable structure/property relationships and optimal material design paradigms. While reducing the injection barrier and contact resistance remains critical for achieving high OFET performance, this work demonstrates a path toward consistently achieving high charge carrier mobility through device geometry design, ultimately reducing processing complexity and cost.

## Graphical abstract

Large‐scale simulations identify device design parameters to circumvent charge injection limitations in organic field‐effect transistors, enabling high charge carrier mobility (>5 cm^2^ V^−1^s^−1^) in all‐organic, solution‐processed devices with printed contacts.

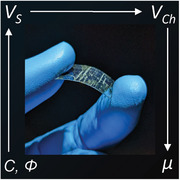

## 1 Introduction

Organic semiconductors (OSC) are at the forefront of many emerging technologies like flexible displays, custom‐printed circuits, and conformable biosensors.^[^ ^1^, ^2^, ^3^, ^4^ ^]^ One major bottleneck is the inefficient charge injection from electrodes into the OSC layer, leading to contact resistance (*R_C_*).^[^ ^5^, ^6^, ^7^, ^8^, ^9^, ^10^, ^11^ ^]^ Extensive research has yielded solutions like electrode modification with self‐assembled monolayers (SAMs),^[^ ^5^, ^12^, ^13^ ^]^ insertion of interlayers,^[^ ^5^, ^6^, ^7^ ^]^ or doping,^[^ ^14^, ^15^, ^16^ ^]^ leading to high‐performance devices.^[^ ^2^, ^16^, ^17^, ^18^, ^19^, ^20^, ^21^ ^]^ However, replicating these successes with low‐cost, scalable manufacturing remains a challenge.^[^ ^2^, ^9^, ^22^, ^23^ ^]^ Organic contacts offer a potential solution, being printable and mechanically flexible, but they often create large injection (Schottky) barriers (*Φ*), leading to poor performance unless the device geometry unintentionally compensates for this issue.^[^ ^24^, ^25^, ^26^, ^27^, ^28^ ^]^ Achieving consistent high mobility in simple device architectures requires minimizing the impact of non‐ideal interfacial processes. This is vital not only for device design but also for material development, as current injection limitations hinder the exploration of fundamental mobility limits of materials. The large number of possible material and device geometry combinations makes this goal extremely challenging.

Here, we employed a data‐driven device design to streamline the search through this vast parameter space and determine the conditions where the contact quality is less consequential for device operation. We focused on organic field‐effect transistors (OFETs), which operate in a high charge‐carrier density regime (10^17^−10^20^ cm^−3^) compared to other organic devices, consequently experiencing significant stress at the contacts.^[^ ^4^, ^5^, ^9^, ^10^, ^19^, ^22^, ^29^, ^30^, ^31^, ^32^, ^33^ ^]^ Through an iterative feedback loop between simulations and experiments, we identified the fabrication windows where device mobility is insensitive to the Schottky barrier. This prediction of barrier‐tolerant OFETs challenges conventional wisdom, but it significantly widens the choice of contact materials, allowing the inclusion of some with less demanding processing. As such, we identified solution‐processable organic electrodes that comply with the window established by simulations and incorporated them in solution‐processed all‐organic OFETs on flexible substrates. Remarkably, these rationally designed devices exhibit text‐book characteristics and charge carrier mobilities exceeding 5 cm^2^ V^−1^s^−1^ without any optimization. Our results establish a reliable pathway to accelerate device optimization by enabling the selection of cost‐effective, scalable contact materials without compromising mobility. This strategy not only guides the selection of geometric parameters essential for the consistent achievement of high mobilities but also facilitates access to the fundamental limits of material efficiency, a critical step in the design and development of novel organic semiconductors.

## 2 Results

### 2.1 Simulation‐Guided Fabrication of Schottky‐Barrier Tolerant OFETs

**Figure 1:**
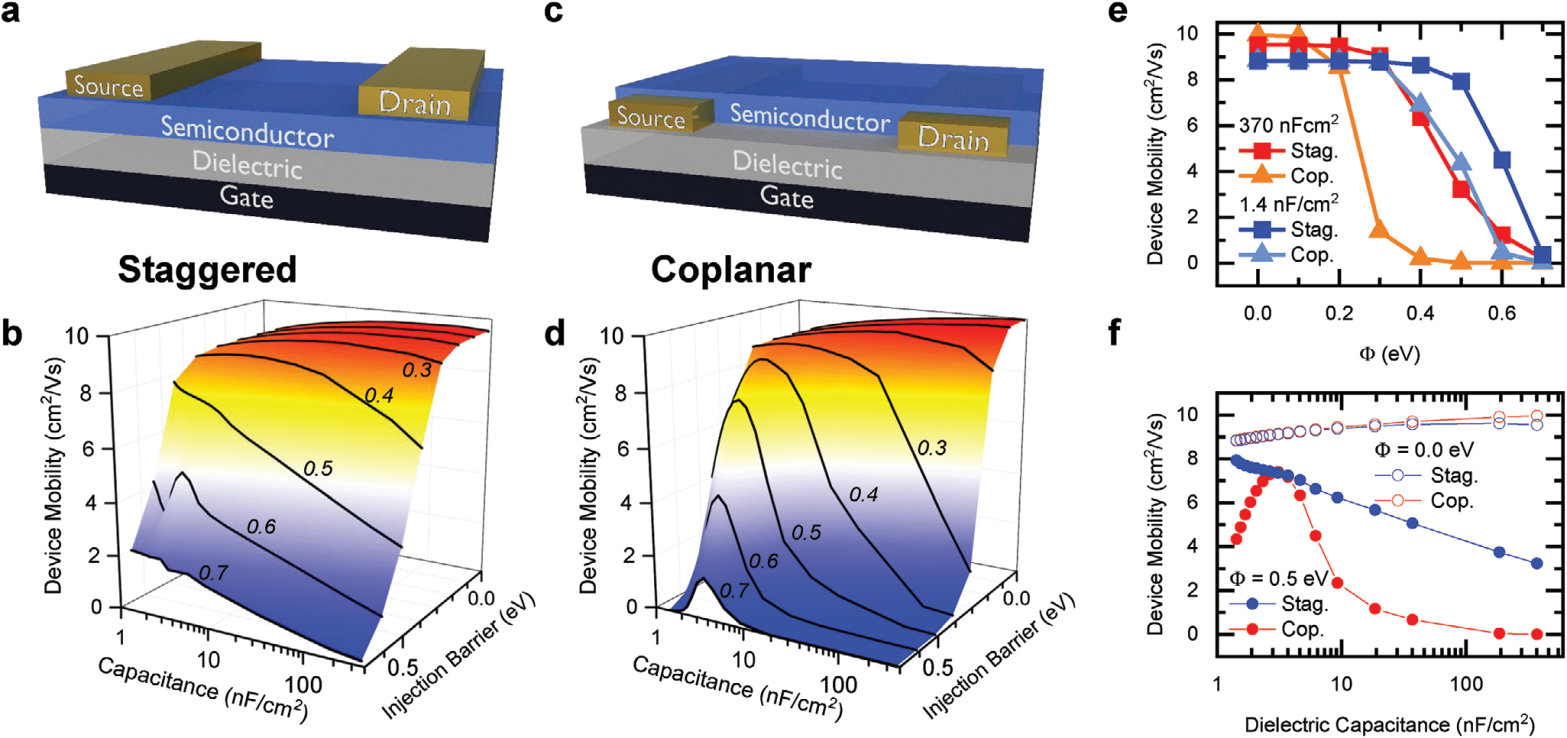
Simulation results for mobility in OFETs with different geometries. a) Staggered OFET structure with top contact, bottom gate configuration. b) Dependence of OFET mobility on the injection barrier (*Φ*), and dielectric capacitance in staggered devices. c) Coplanar devices in the bottom gate, bottom contact configuration. d) Dependence of OFET mobility on the injection barrier (*Φ*), and dielectric capacitance in coplanar devices. e) Mobility as a function of injection barrier for high‐capacitance (*C_high_* = 370 nF cm^−2^, red and orange) and low‐capacitance (*C_low_* = 1.4 nF cm^−2^, blue and light blue) devices, for both staggered (squares) and coplanar (triangles) configurations. f) Device mobility versus dielectric capacitance in staggered and coplanar (blue and red, respectively) for *Φ* = 0 eV and *Φ* = 0.5 eV (open and filled, respectively).

2D numerical simulations were performed to investigate the impact of the injection barrier on OFET mobility for several different device configurations. By varying the geometry (order of layers, layer thickness) and material properties (semiconductor mobility, trap density of states, electrode work function), we investigated over 2000 distinct OFETs, thus creating a vast multi‐dimensional parameter space to examine. The results for an OSC with an intrinsic mobility of *µ_i_* = 10 cm^2^ V^−1^s^−1^ are summarized in **Figure** **1**. We provide corresponding plots for *µ_i_* = 1.6 cm^2^ V^−1^s^−1^ in Figure S1 (Supporting Information), while in Figure S2 (Supporting Information) we show the dependence of device mobility on the injection barrier for different values of semiconductor dielectric constant. Importantly, our analysis reveals that neither changes in the intrinsic mobility of the organic semiconductor, nor its dielectric constant, alter the fundamental nature of the observed behavior, suggesting that our findings are broadly applicable to a wide range of organic semiconductors.

Example calculated *I–V* curves are displayed in Figure S3 (Supporting Information). The top contact, bottom gate geometry (Figure 1a) represents the staggered structure,^[^ ^5^, ^22^, ^23^ ^]^ for which Figure 1b reveals several trends between device mobility *µ*, injection barrier *Φ*, and dielectric capacitance *C* (solid lines represent curves of constant *Φ*). First, for small injection barriers, *Φ *≤ 0.2 eV, *Φ* and *C* are predicted to not have a significant effect on *µ* (the spread in mobility is lower than 7%). Second, when *Φ* > 0.2 eV, *µ* decreases with increasing *Φ*, as expected.^[^ ^7^, ^12^, ^34^ ^]^ Third, and most intriguing, is that for moderate injection barriers, 0.2 eV ≤ *Φ* ≤ 0.5 eV, *µ* can be increased by lowering *C*. A substantial drop in mobility is predicted for very large injection barriers, *Φ* > 0.5 eV, even for the low‐*C* devices, defining the upper boundary for the fabrication window that yields a high‐mobility. The predicted *µ–C* dependence is more significant than previously thought,^[^ ^8^, ^34^ ^]^ given that our simulations were carried out using a much wider range of injection barriers and capacitances (*Φ* = 0.0 eV to 0.7 eV, *C* = 1.4 to 372 nF cm^−2^) and due to the fact that a more realistic trap density of states was programmed into the bulk semiconductor properties by replacing the typical trap‐free system with an experimentally‐derived trap density of states (t‐DOS).^[^ ^33^, ^35^ ^]^ More details on the calculations of t‐DOS are included in the Experimental Section and Figure S4 (Supporting Information). Notably, our calculations have the flexibility to incorporate variations in the trap density of states environment, enabling a more accurate reflection of diverse experimental conditions.^[^ ^36^ ^]^ The second geometry simulated was the coplanar structure (Figure 1c).^[^ ^5^, ^22^, ^23^ ^]^ The observed trends are similar to those found in the staggered structures, but now *µ* exhibits a more pronounced dependence on *C*, with a notable decrease in the very low‐*C* regime (Figure 1d). This reduction in *µ* is attributed to the increased sensitivity of coplanar devices to the gate field strength at the injection, as established in previous studies.^[^ ^5^, ^34^ ^]^ Consistent with this interpretation, we observe a corresponding decline in the tunneling rate near the injection contact, mirroring the trend in mobility (Figure S5, Supporting Information).

Slices were taken at constant capacitance in Figure 1b,d, and the results are shown in Figure 1e, where mobility is plotted versus the injection barrier for high‐ and low‐capacitance devices, in staggered and coplanar geometries. The mobility is independent of *Φ* at low injection barriers, then its decrease occurs at different critical *Φ* values, ranging from as low as *Φ* = 0.2 eV for the high‐*C*, coplanar devices to *Φ* = 0.5 eV for the low‐*C*, staggered devices. This prediction is extremely valuable because it could provide the much‐sought path to achieving high‐mobility devices with significantly less manufacturing constraints by providing a 0.3 eV larger injection window (0.2 eV ≤ *Φ* ≤ 0.5 eV) and thus greatly broadening the library of compatible contact materials. By plotting the mobility versus capacitance at a constant injection barrier (Figure 1f), it is evident that in ideal transistors (*Φ *= 0 eV) the device architecture (i.e., capacitance, layer order) has negligible effects on the performance. However, when the barrier is present (*Φ* = 0.5 eV), the mobility exhibits a strong dependence on the capacitance, and the staggered structure is superior to the coplanar structure, retaining higher mobility throughout the capacitance range, and within 10% of the *Φ* = 0 eV devices at *C* = 1.4 nF cm^−2^. In summary, the simulation results cumulatively predict that the staggered structure is more tolerant of an injection barrier and that when the barrier is present, device mobility can be increased by reducing the dielectric capacitance.

### 2.2 Recovery of Device Performance in the Presence of Schottky Effects

To experimentally verify the theoretical predictions, we fabricated OFETs based on the polymer semiconductor indacenodithiophene‐*co*‐benzothiadiazole (IDT‐BT) in a variety of device structures.^[^ ^13^, ^29^, ^37^, ^38^, ^39^, ^40^, ^41^ ^]^ First, we confirmed the lower sensitivity to the presence of an injection barrier for the staggered structures by fabricating devices with staggered contacts with *Φ* = 0.9 eV and coplanar contacts of *Φ* = 0.4 eV on the same dielectric wherein the staggered devices outperformed the coplanar devices in spite of the less advantageous injection barrier (Figure S6, Supporting Information). With that result in mind, for subsequent investigations, we focused on staggered devices with top gate, bottom contact geometry. Gold source and drain contacts were treated with a pentafluorobenzene thiol (PFBT) SAM or left untreated, resulting in estimated injection barriers of 0 to 0.4 eV, respectively. It is important to recognize that these values are derived from the difference between the contact work function and the HOMO level of IDT‐BT. While these estimates may not represent the true injection barriers, they nevertheless offer a quantifiable metric for systematically modulating the injection process.^[^ ^5^, ^42^ ^]^ To vary the capacitance, the dielectric thickness was modified while maintaining the same dielectric layer (Cytop) for all devices to minimize the contributions of additional phenomena like charge scattering due to roughness,^[^ ^43^ ^]^ Fröhlich polarons,^[^ ^44^ ^]^ or interfacial trap density.^[^ ^33^ ^]^ This resulted in a narrower capacitance range (*C* = 1.6 to 5.0 nF cm^−2^) than the one scanned in the simulation efforts. However, even within this restricted window, we observed significant changes in mobility, which were successfully captured and explained by our simulations. We acknowledge the greater change in the experimental results compared to the predicted 14% mobility drop when scaling from 1.6 to 3.7 nF cm^−2^ in devices with a 0.5 eV injection barrier. This suggests the need for a more comprehensive model that incorporates a wider array of parameters to describe the organic semiconductor layer properties, such as the distribution of trap states, static and energetic disorder, for a direct comparison of the simulation and experimental results at a specific capacitance value. Although such a direct quantitative comparison remains challenging, the qualitative agreement between observed and predicted trends provides strong support for the validity of our approach.

**Figure 2:**
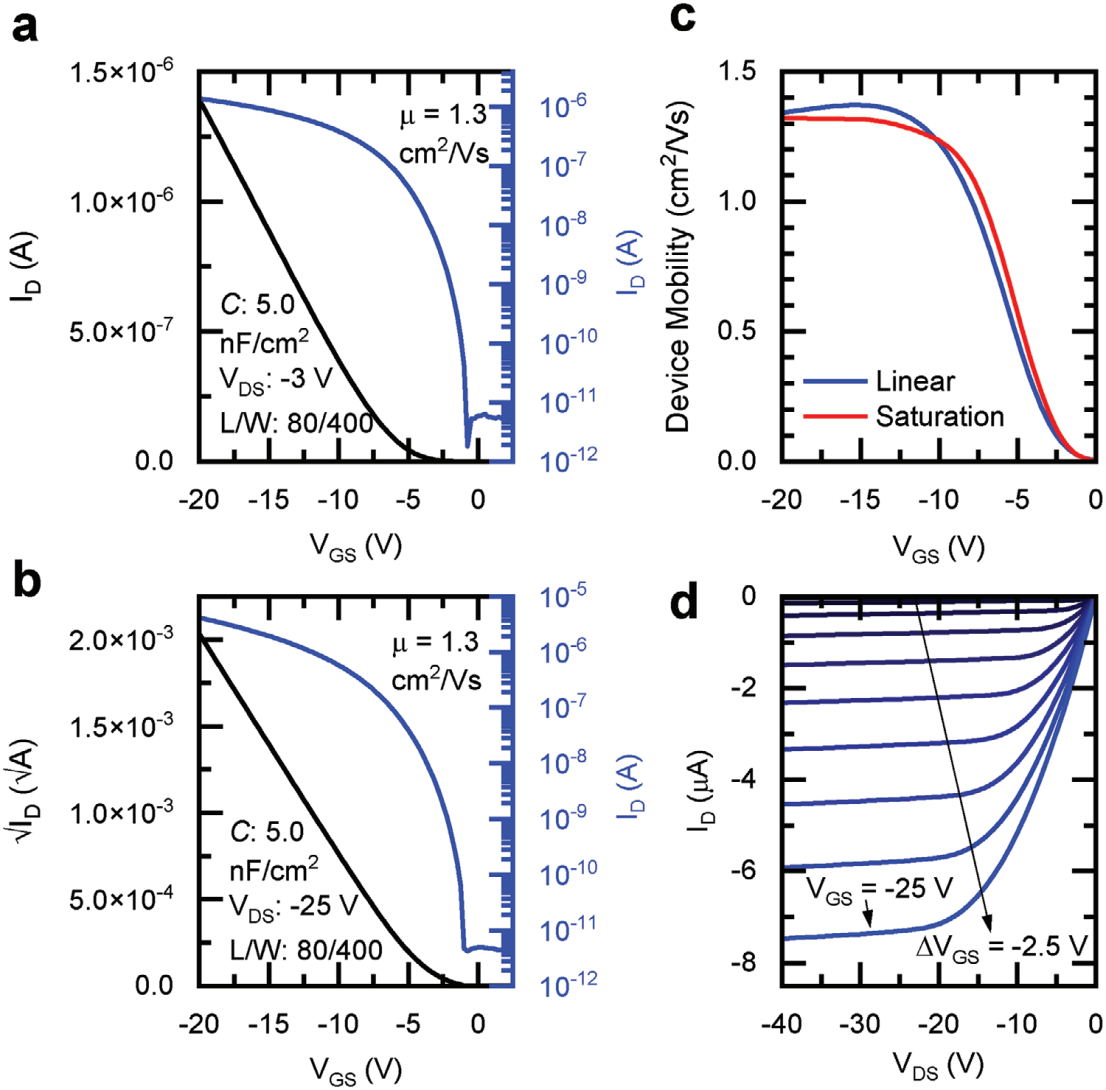
Device performance of experimental OFET based on IDT‐BT. a) *I–V* curve in the linear regime obtained in a transistor with high capacitance (*C* = 5.0 nF cm^−2^) and low injection barrier (*Φ* = 0 eV). b) Corresponding *I–V* curve in the saturation regime. c) Linear and saturation mobility as a function of gate voltage extracted from the *I–V* curves in panels *a* and *b*. d) Output characteristics of the same device.

Example linear and saturation transfer curves (drain current *I_D_* vs gate‐source voltage *V_GS_*) for a typical OFET (*C* = 5.0 nF cm^−2^, *Φ* = 0 eV) are displayed in **Figure** **2**a,b, respectively – examples for other combinations of *C* and *Φ* can be found in Figure S7 (Supporting Information). These curves show no evidence of the “double‐slope,”^[^ ^30^, ^45^, ^46^, ^47^ ^]^ have turn‐on voltages of −1 V or less, and an extremely steep turn on, with subthreshold slopes (STS) of 250 mV dec^−1^, the smallest in an IDT‐BT transistor.^[^ ^38^ ^]^ The linearity in the transfer curves results in a mobility that is independent of the gate voltage, while the low contact resistance yields similar linear and saturation mobility (Figure 2c). Output characteristics (*I_D_* vs drain‐source voltage *V_DS_*, Figure 2d) also align with expectations for an ideal device, devoid of the “s‐shape” that signifies contact resistance.^[^ ^5^ ^]^

**Figure 3:**
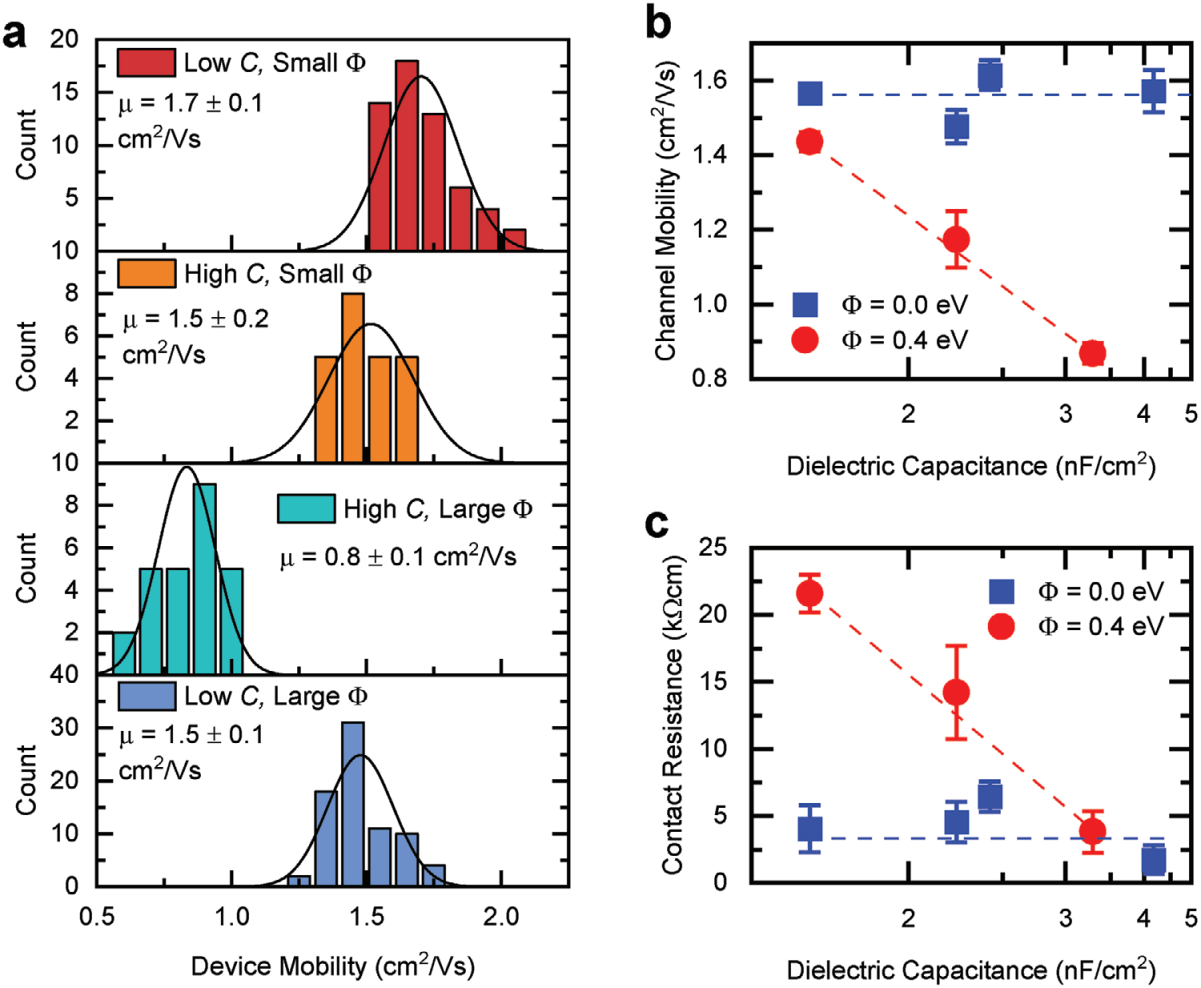
Mobility extracted from experimental OFETs with varying capacitance and injection barrier. a) Histogram of mobilities for four device types: combinations of low *C* = 1.6 ± 0.1 nF cm^−2^, high *C* = 3.7 ± 0.2 nF cm^−2^, small *Φ* = 0.0 eV, and large *Φ* = 0.4 eV. A minimum of 25 devices of each type have been tested and the results have been included in these graphs. b) Device mobility as a function of dielectric capacitance, for devices with *Φ* = 0 eV (blue) and *Φ* = 0.4 eV (red); lines are guides for the eye. c) Contact resistance as a function of dielectric capacitance, as calculated by gated‐TLM; lines are guides for the eye.

Mobility histograms corresponding to OFETs representing four different *C*–*Φ* combinations are shown in **Figure** **3**a: here the high‐*C* dielectric refers to *C* = 3.7 ± 0.2 nF cm^−2^ and low‐*C* to *C* = 1.6 ± 0.1 nF cm^−2^, while small/large *Φ* correspond to 0 and 0.4 eV, respectively. All OFET arrays exhibited an extremely high degree of uniformity as demonstrated by the narrow spread in mobility. For the barrier‐free OFETs (top two panels), the average mobilities of the low‐*C* and high‐*C* devices were comparable within the measurement accuracy: 1.7 ± 0.1 and 1.5 ± 0.2 cm^2^ V^−1^s^−1^, respectively, in agreement with the predictions in Figure 1. When the large injection barrier was introduced, however, the mobility dropped by about a factor of two to 0.8 ± 0.1cm^2^ V^−1^s^−1^ for high‐*C* devices. On the contrary, in the low‐*C* transistors, we recovered the performance of the devices with ohmic contacts, with an average mobility of 1.5 ± 0.1 cm^2^ V^−1^s^−1^. We tested devices with channel lengths between 30 and 100 µm and observed that the mobility remained unaffected by channel length (Figure S8, Supporting Information). This indicates the potential for even further downscaling without compromising performance. While this is remarkable given the common expectations that an injection barrier impedes a device from reaching a high performance,^[^ ^7^, ^12^, ^13^, ^48^ ^]^ it is now not surprising given that this geometry lies in the injection‐tolerant window identified by us using simulations. We incorporated the results presented so far, alongside data gathered from additional devices with other values of *C*, and plotted the experimentally‐derived device mobility versus dielectric capacitance for two barrier types in Figure 3b. As expected, the mobility of the *Φ* = 0 eV devices is constant, while that of the devices where an injection barrier is present (*Φ* = 0.4 eV) drops with increasing *C*. This result experimentally validates the fact that at sufficiently small *C* the high mobility is restored in OFETs with a high Schottky barrier at the electrode/semiconductor interface.

To investigate why the injection barrier causes a drop in mobility in high‐*C* devices but not in low‐*C* devices, we calculated *R_C_* using the gated‐transfer length method (gTLM – see Figure S9, Table S1, Supporting Information).^[^ ^5^, ^11^, ^22^, ^49^ ^]^ The width‐normalized contact resistance (*R_C_W*) and channel resistance per unit length (*R_Ch_W/L*) are given by the intercept and slope, respectively, of a linear regression of the total resistance (*R_tot_W*) versus *L*. The trend of lowering *R_C_* by increasing *C* is present for the devices with the injection barrier, in agreement with previous reports,^[^ ^50^, ^51^ ^]^ implying that the high mobility in the low‐*C* devices is obtained in spite of less efficient injection (Figure 3c). Therefore, contact resistance alone cannot explain the discrepancy in mobility in devices with *Φ* = 0.4 eV. To compare channel effects normalized for *C*, we converted *R_Ch_W* to channel mobility (*µ_ch_*), which represents the device mobility in the absence of contact effects. These values mirror the dependence of the device mobility, providing evidence that the reduced channel mobility in high‐*C* OFETs with large *Φ* is responsible for lowering the device mobility. Fortunately, we can now suppress this effect and create barrier‐tolerant OFETs by designing low‐capacitance devices.

**Figure 4:**
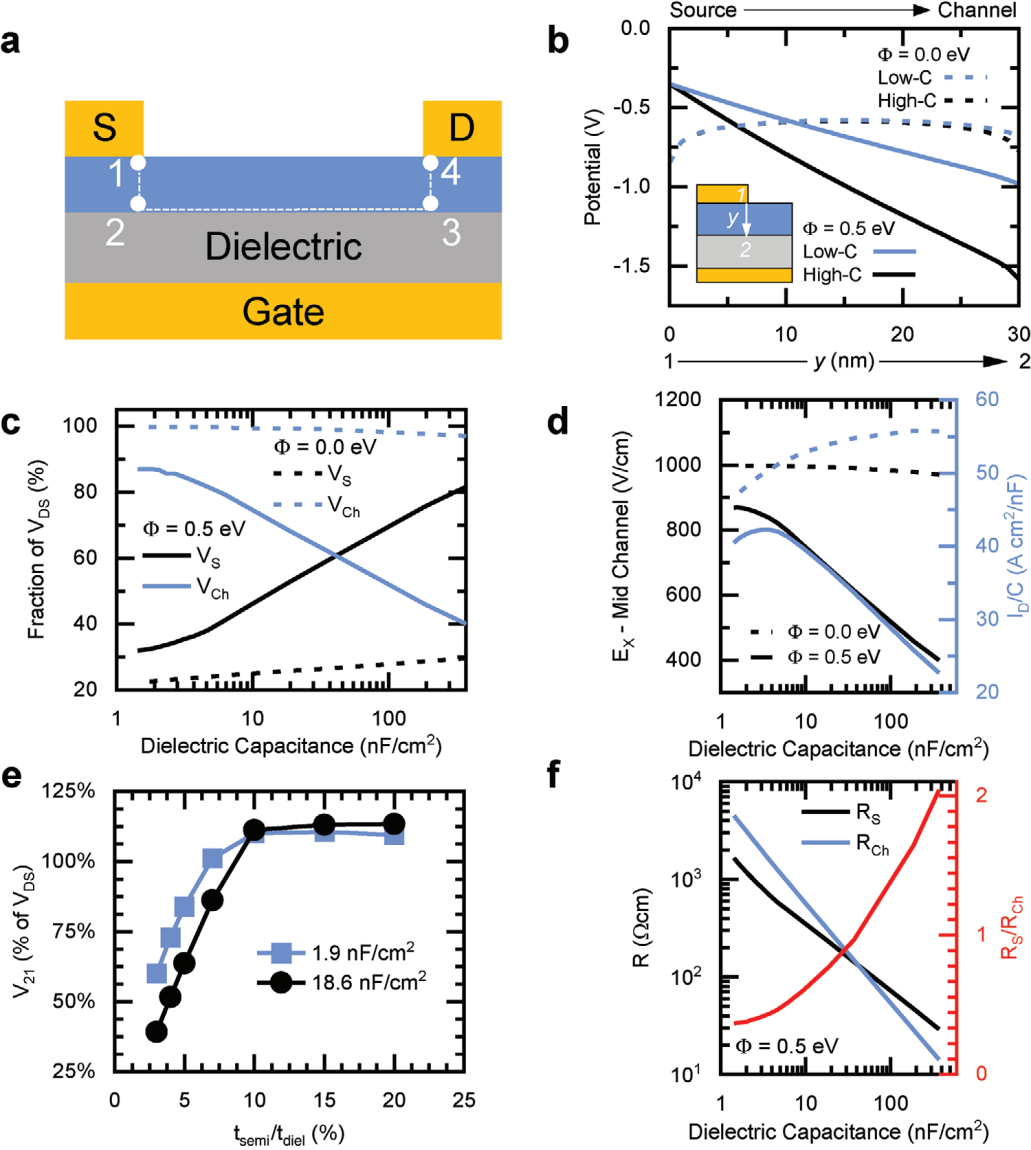
a) Points of virtual probes to measure potential (“1” to “4”) and path of charge transfer (white line). b) Electric potential along the injection path (line from Point 1 to Point 2) for several *C*, *Φ* combinations. c) Source contact voltage *V_S_* ≡ *V_21_* and channel voltage *V_Ch_* ≡ *V_32_* as a fraction of *V_DS_* for *Φ* = 0.0 eV and *Φ* = 0.5 eV. d) Electric field strength parallel to the channel (black, left) and corresponding *I_D_* normalized by *C* (blue, right). e) Source contact voltage *V_S_* ≡ *V_21_* as a function of the ratio of the thickness of the semiconductor layer (*t_semi_*) to the thickness of the dielectric layer (*t_diel_*). f) Contact resistance (black, left), channel resistance (blue, left), and the ratio of contact to channel resistance (red, right) as a function of C.

The influence of the injection barrier on channel mobility might appear peculiar since *µ_ch_* is primarily associated with the semiconductor/dielectric interface, but it can be explained considering the relationship between the electric field associated with charge injection, modulated by *Φ* and *C*, and the electric field in the conduction channel, which directly impacts device mobility. Our comprehensive simulations have enabled the exploration of this causal relationship (refer to Figures S10–S20, Supporting Information for further insights). **Figure** **4**a displays the path of charge carriers (dotted white line): a line from point 1, the source contact, to point 2, the start of the channel represents the injection process. The channel is point 2 to point 3, and charge collection occurs between point 3 and point 4, the drain contact.

The simulated electric potential along the injection path through the 30 nm‐thick semiconductor voltage between points 1 and 2 is plotted in Figure 4b, that is, the path from the source contact and to the start of the channel, for several combinations of *C* and *Φ* (low‐*C* = 1.9 nF cm^−2^ and high‐*C* = 18.6 nF cm^−2^). For an ideal contact, the injection process is bulk‐limited in the semiconductor layer, and a space charge forms near the contact (Figure S13, Supporting Information); the space charge screens the gate field and the result is a negligible potential drop along the injection path from source to channel (point 1 to point 2) as shown in Figure 4b (dotted lines). On the other hand, in the case of contact‐limited injection, charges must tunnel through or around the energy barrier arising from the Schottky contact; there is insufficient injection to build up charge in the semiconductor bulk, and so there is no space charge screening the gate field. The result is a non‐negligible voltage drop from the source contact to the channel, that is, |*V_S_* | > 0, as shown in Figure 4b (solid lines).

To examine the effect of *C*, consider that the gate field is distributed across both the dielectric and semiconductor layers, similar to how voltage is distributed across two capacitors in series. If *C* is small relative to the capacitance of the semiconductor layer, meaning the dielectric is much thicker than the semiconductor film, then the dielectric layer dominates the share of the gate field. Conversely, for a high‐capacitance dielectric, a large potential drop across the semiconductor layer occurs, which is equivalent to a |*V_21_* | increase. In other words, the source contact voltage *V*_S_ ≡ *V_21_* scales with *C* as shown in Figure 4c; because of the lack of charge screening in devices with a large injection barrier (solid lines, *Φ *= 0.5 eV), the dependence of *V_S_* on *C* is most prominent in these versus devices with ideal contacts (dotted lines, *Φ* = 0.0 eV).

The applied drain‐source voltage *V_DS_* is shared across the source contact, the channel, and the drain (*V_DS_* = *V_S_* + *V_Ch_* + *V_D_*, with *V_ch_* ≡ *V_32_*, *V_D_* = *V_43_*), and so when *V_S_* increases, then *V_ch_* must decrease (*V_D_* is much smaller than *V_S_* or *V_ch_*). This is depicted in Figure 4c: when *V_S_* increases with *C* (blue), *V_ch_* decreases (black). In other words, the electric field in the channel that is associated with charge mobility is reduced with increasing *C*, which corresponds with a smaller drain current *I_D_* and reduced device mobility (Figure 4d).

Though the discussion above focused on manipulating *C*, we emphasize that changing the thickness of the semiconductor layer will have a similar effect on device performance since changing layer thickness will alter the distribution of the gate field across the semiconductor and dielectric. Figure 4e demonstrates that |*V_21_* | scales as the ratio of the thickness/capacitance of the semiconductor and dielectric layers, regardless of the value of *C*. Therefore, when downscaling devices in *C*, care must be taken such that an unintended loss of device mobility does not occur.

An important consideration is that contact resistance scales inversely with *V_S_* for a given value of *Φ* (Figure 4f). However, the higher‐*C* devices have lower source‐contact resistance *R_S_* since the larger *V_S_* is offset by even larger gains in *I_D_*. In fact, the strength of the injection field from the large value of *V_S_* in a high‐*C* device aids charge injection, but at the cost of *V_ch_*. The ratio between *R_S_* and *R_Ch_* determines if an OFET is contact‐dominated, and the ratio increases much more rapidly with *C* when an injection barrier is present, in which case a low‐*C* device is favorable.

In summary, when an OFET has an injection barrier, then charge injection requires a non‐negligible voltage taken from the applied *V_DS_*, which in turn reduces the electric field strength in the channel and thus the device mobility. The magnitude of this effect is modulated by *C*, wherein adopting a low‐capacitance design alleviates the effect and device performance can be restored to nearly that of a device with ideal contacts.

### 2.3 Roadmap for All‐Organic, All‐Solution Processed Transistors

The results guided by simulation and confirmed by experiment concurrently show that when the capacitance of the dielectric is sufficiently small (≈1 to 2 nF cm^−2^) there is a window where the injection barrier is not significantly impacting device mobility, namely from 0 to 0.5 eV. This generous range significantly expands the choice of electrode material by enabling the selection of compounds that are more attractive from the standpoint of processing but have been previously dismissed. One candidate is poly(3,4‐ethylenedioxythiophene) polystyrene sulfonate (PEDOT: PSS), a conducting polymer blend with a work function of ≈5.0 eV that can be printed and is nearly transparent in thin layers.^[^ ^31^, ^52^, ^53^ ^]^ The PEDOT: PSS/IDT‐BT interface creates a 0.4 eV injection barrier (if additional interfacial effects are ignored), putting the system within the processing window identified in this work.

**Figure 5:**
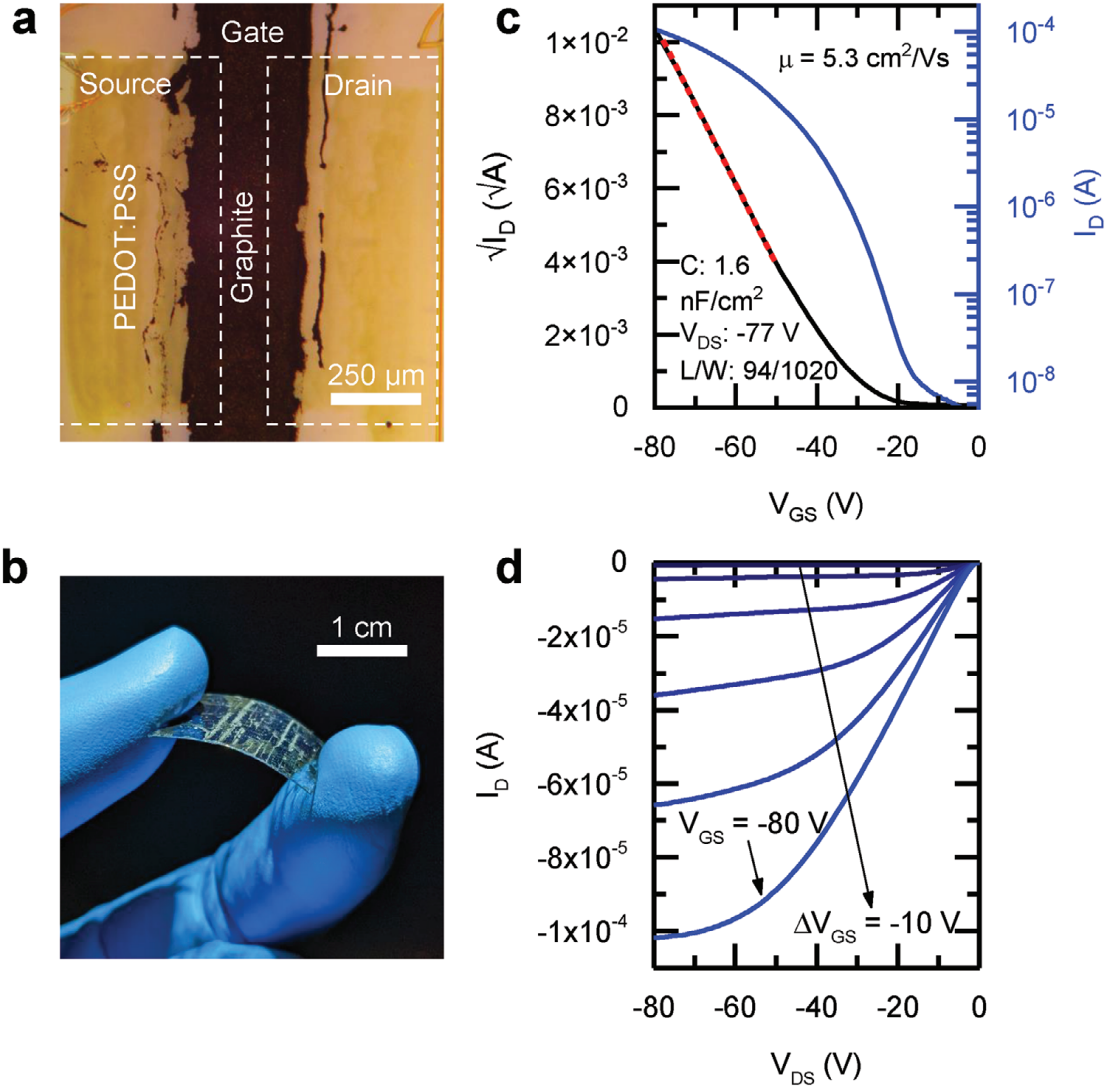
Solution‐deposited high‐performance organic transistors on flexible substrates a) Micrograph of an OFET with printed PEDOT: PSS source/drain contacts, Cytop dielectric, and graphite gate electrode. Note that the PEDOT: PSS is nearly transparent. b) OFET device arrays on a PET substrate. c) *I–V* curves in the saturation regime for the device displaying the highest mobility of the PEDOT: PSS set, 5.3 cm^2^ V^−1^s^−1^. The dotted red line represents the segment of the *I–V* curve from which mobility was calculated. d) Corresponding output *I–V* curves of the same device taken from *V_GS_* = 0 V to *V_GS_* = −80 V.

For proof of concept, we fabricated fully organic, all‐solution processed OFETs with printed PEDOT: PSS source and drain contacts and sprayed graphite gate electrodes on flexible substrates. Subsequently, we compared their performance with that of the most optimized conventional devices presented earlier (**Figure** **5**a,b – see also Figure S21, Supporting Information). The transfer and output curves for a device with *C* = 1.6 nF cm^−2^ are shown in Figure 5c,d; similar to the OFETs with PFBT/Au electrodes, the *I–V* curves are of high quality, without double‐slopes or peaks in mobility as a function of gate voltage (Figure S22, Supporting Information). Remarkably, we recorded a system average device mobility of 3.5 ± 1.1 cm^2^ V^−1^s^−1^, with the best device measuring *µ* = 5.3 cm^2^ V^−1^s^−1^. These values, which represent device mobilities, not corrected for contact effects, are on par with the values obtained on PFBT/Au contacts and significantly higher than any previously reported fully‐solution‐deposited OFETs.^[^ ^25^, ^26^, ^53^ ^]^ In fact, a small improvement in mobility is observed compared to devices with PFBT/Au contacts. This phenomenon, where organic contacts outperform inorganic contacts with equivalent functions, has been reported for other systems and attributed to enhanced charge injection due to interactions between the copolymer's acceptor/donor units and the charge‐transfer organic contacts, as well as enhanced semiconductor film quality near the interface.^[^ ^24^, ^27^, ^28^ ^]^ Our devices with PEDOT: PSS contacts and low‐*C* dielectric approach the performance of the best OFETs made with IDT‐BT,^[^ ^13^, ^37^ ^]^ but at a fraction of the cost. These results are remarkable because the high‐mobility devices have been developed following the simulation‐guided design strategy developed in this study, with no additional optimization.

Not only have we eliminated the low‐yield, resource intense trial and error approach, and in doing so significantly accelerated the progress, but we devised a strategy for incorporating a wider material set in device architectures – a significant step in the development of large area, low‐cost printed OFET arrays on flexible substrates. The performance of OFETs with contacts consisting of materials with work functions laying outside of the injection‐tolerant window has also been tested (Figure S23, Supporting Information). These measurements confirm that the high‐mobility devices are restricted to the fabrication window projected by the simulations, and when the injection barrier exceeds the maximum boundary (and the contact resistance is too high), adopting the low‐*C* design is not sufficient to recover the performance.

**Figure 6:**
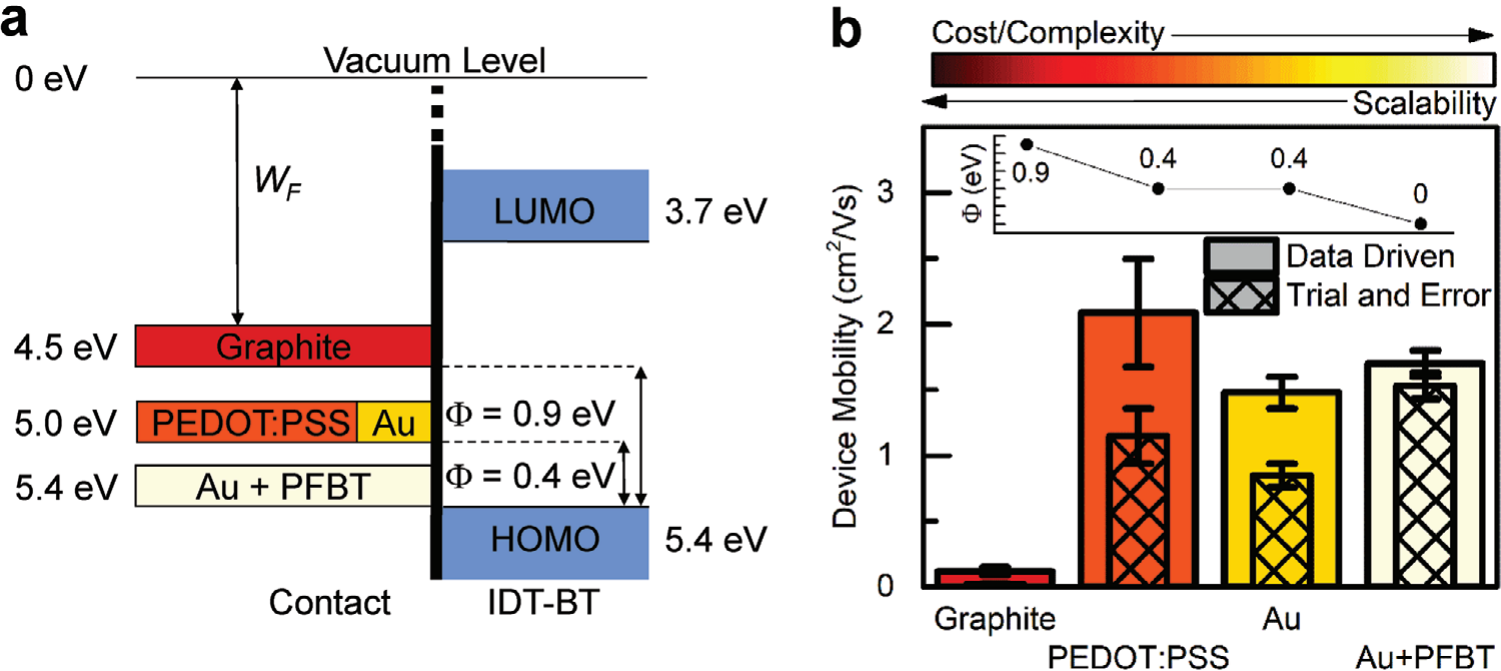
Low‐cost, high‐performance transistors with scalable processing achieved by optimization strategy driven by simulation‐guided design. a) Tested contact materials and their work functions in relation to the HOMO/LUMO levels of IDT‐BT. The band bending is ignored for simplicity. b) Experimental device mobility of OFETs with various source/drain contacts and Au gate electrode: “Data‐Driven” designs (solid color) and reference devices that have a dielectric capacitance outside the fabrication window, “Trial and Error” (crosshatched inset bars). The inset displays the injection barrier for each contact type. Color scale corresponds to the cost/complexity of fabrication, which is inversely related to its scalability.

A summary of the experimental results obtained on the four different types of source/drain electrodes tested is shown in **Figure** **6**: in Figure 6a we sketch the energy level alignment between the contact work function and the organic semiconductor highest occupied molecular orbital (HOMO) level, while the corresponding OFET mobility is included in Figure 6b. Here, the geometries within the injection‐tolerant window, and hence corresponding to the data‐driven transistor design, are labeled as “data driven”, while those outside this window are referred to as “trial and error”. The contact resistance values extracted from gated‐TLM showed good agreement with the injection barrier estimated from the work function of each of the four different contact types (Table S2, Supporting Information). It is important to acknowledge that interfacial phenomena such as energy‐level bending, morphological variations, trap states, and spontaneous dipoles can influence contact behavior and may contribute to discrepancies between different interfaces.^[^ ^5^, ^42^ ^]^ With the trial‐and‐error devices (crosshatched inset bars), there is a substantial trade‐off between the cost/complexity of the contact engineering and the resulting performance. For instance, when contacts which induce a high barrier (0.2 ≤ *Φ* ≤ 0.5 eV), such as the case of PEDOT: PSS or Au, are used for semiconductor prototyping in a non‐optimal OFET geometry, device characterization yields an underestimation of the material mobility and additional processing steps (e.g., chemical functionalization of the electrodes) are necessary in order to access the fundamental limits. These steps can substantially add to the fabrication costs, are restricted to traditional metals (Au, Ag, Cu, etc.), and only apply to bottom‐contact geometries.

In contrast, the OFETs complying with the geometries identified by the data‐driven approach are not subject to the same trade‐offs and consistently exhibit a high device mobility regardless of the contact material used, providing a path for device design where cost and performance are not mutually exclusive. Indeed, OFETs with printed PEDOT: PSS contacts (*Φ* = 0.4 eV) exhibit mobilities on par with those obtained in OFETs with Au+PFBT contacts (*Φ* = 0 eV); all barrier heights are calculated from the difference in the work function of the electrode and the HOMO level of the organic semiconductor. While these values provide a useful estimation, they may not perfectly reflect the actual injection barriers present in the devices due to the complex interplay of interfacial phenomena. An added advantage is that the reduced restrictions in electrode choice open new avenues into materials exploration and allow consideration of solution‐processable organic materials like PEDOT: PSS, which bring several assets. First, they are processed by scalable and low‐cost methods and thus could potentially close the manufacturability‐scalability‐sustainability‐performance gaps, a key requirement for the integration of organic semiconductors into next‐generation optoelectronic devices. Second, exploiting the benefits of the organic/organic interface without suffering from the low work function penalty may lead to consistent access of the intrinsic properties of the organic semiconductor, an essential step in rational material design.

## 3 Conclusion

In summary, we used simulations to identify the fabrication window wherein the OFET mobility is decoupled from the work function of the contact and then exploited this knowledge to guide the design of low‐cost, high‐mobility devices on flexible substrates for which all layers are solution‐deposited. By using simulations to guide our design, we were able to virtually explore over 2000 different device configurations. This efficient approach significantly reduced the need for physical prototypes, requiring only a few experimental devices to validate our predictions and confirm our hypothesis. The success highlights the value of this design process, which considers a wide range of device parameters and identifies optimal configurations that achieve high mobility irrespective of the Schottky barrier height at the electrode/organic semiconductor interface. We experimentally validated our predictions in OFETs with conventional structures and determined that the maximum barrier height recoverable through dielectric capacitance reduction is ≈0.5 eV. OFETs with a low‐capacitance dielectric (1.6 nF cm^−^^2^) and a 0.4 eV injection barrier achieved mobility comparable to devices with negligible injection barriers. We explained the results by considering the net electric field distribution in the device channel and found that the low‐capacitance dielectric preserves the electric field strength in the channel and hence the device mobility. By pinpointing the critical elements of barrier‐tolerant OFET architectures, we were able to successfully adapt our fabrication process to create all‐solution‐processed transistors on flexible substrates. These devices yield mobilities exceeding 5 cm^2^ V^−1^s^−1^ – the highest obtained so far in all‐solution‐processed OFETs.

The impact of our work is threefold: first, it provides a methodology that enables access to mobilities approaching the intrinsic limit even in the presence of moderate injection barriers. This outcome will significantly impact the material design by facilitating rigorous evaluation of material structure–property relationships, leading to accurate feedback for material design efforts. Second, it delineates material and geometry windows that permit the utilization of solution‐processable materials for all device layers without compromising on final performance, thus truly exploiting the touted potential of organic devices. Third, we established a framework for material processing and device design through the effective use of high‐throughput simulations, replacing the traditional trial‐and‐error approaches. Notably, these results are not material dependent, so we expect that similar trends apply to other small molecule and polymeric organic semiconductors, albeit different boundaries for the fabrication window might apply.

While our model offers valuable insights into the fundamental interplay between injection barriers, dielectric capacitance, and device mobility, it is essential to acknowledge the inherent complexities associated with practical OFET fabrication, which can influence the ultimate device performance. The proposed approach to mitigate the impact of injection barriers presents several technical challenges associated with the reduction of dielectric capacitance. These include the requirement for elevated operating voltages, a reduction in transconductance (*g_m_* – Figure S24, Supporting Information), and an increase in sub‐threshold slope (STS – Figure S25, Supporting Information). Nonetheless, our analysis revealed that for injection‐limited OFETs, the transconductance exhibits a less pronounced reduction in OFETs with capacitances below 10 nF cm^−2^. Furthermore, a substantial reduction in capacitance by a factor of 260 translates to only a modest 22% increase in the sub‐threshold slope. Consequently, embracing contact resistance in the design of organic devices remains a viable strategy for two distinct purposes: 1) evaluating the potential of novel materials and 2) developing low‐frequency applications where such performance parameters are sufficient and the simplified fabrication process offers significant practical advantages, including easier circuit design and implementation. It is essential to reiterate the importance of minimizing contact resistance for pushing the frequency limits of organic circuits, which remains a critical objective.

## 4 Experimental Section

### Simulations

Physically‐based, numerical TCAD simulations were performed with Silvaco Atlas. The simulations were carried out on a Beowulf‐style computer cluster running 12 parallel processors per calculation, which significantly reduced the total computation time. Each calculation used over 200 000 finite element analysis connections for a dense and accurate solution. The transistor geometry was either bottom gate, bottom contact for coplanar devices or bottom gate, top contact for staggered devices (BGTC). Channel length was set to 30 µm and all calculated current values were normalized to a 1000 µm channel width (calculations are 2D and normalized to width). Bias conditions were *V_DS_* = −3 V with source grounded and *V_GS_* swept from 0 to −60 V. A drift‐diffusion model was used for modeling charge transport, while the Universal Schottky Tunneling model was selected for charge injection at the contact/semiconductor interface with surface recombination enabled. The injection barrier was varied from 0.0 to 0.7 eV, and the dielectric capacitance ranged from 1.4 to 372 nF cm^−2^ by modifying the dielectric thickness from 5 to 1300 nm while using a relative dielectric constant of *ɛ* = 2.1. To facilitate accurate modeling, semiconductor regions necessitated a well‐defined band structure. This entailed specifying key parameters such as charge carrier mobility, dielectric constant, electron affinity, and band gap (i.e., HOMO/LUMO levels). Additional material parameters such as the trap density of states could be programmed into the material. The semiconductor LUMO and HOMO levels were set to −3.7 and −5.4 eV, respectively, and an experimentally‐extracted trap density of states as a function of energy above the valance band (*N*(*E*)) was used for the semiconductor bulk.

The trap‐DOS was extracted using the Grünewald method, which treat the *I–V* curve in the linear regime as a measurement of channel conductivity as a function of applied bias. Gate voltage was converted into channel potential, *U_GS_*, by subtracting the turn‐on voltage (which approximates the flat‐band voltage), such that.

(1) $$U_{GS}=V_{GS}\;-\;V_{ON}$$

Ohm's Law was then used to write the channel conductivity (*σ*) as a function of applied bias as

(2) $$\;\sigma \left(U_{GS}\right)=\frac{L}{W}\;\frac{I_{D}}{V_{DS}}$$

Next, this channel conductivity function was used to calculate the channel interface potential (*V_0_*), which resulted from applied bias, through numerically solving

(3)

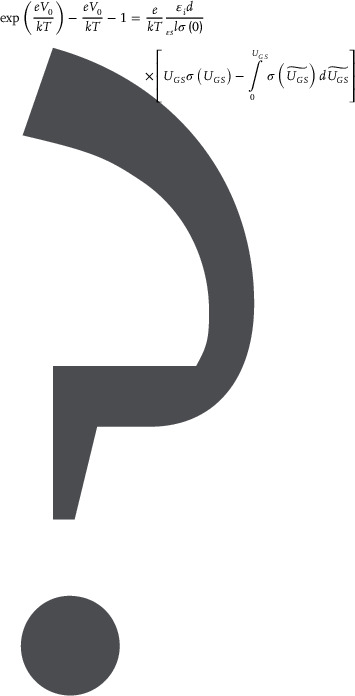

for each bias step in the *I–V* curve. Here, *ɛ_i_* and *l* are the permittivity and thickness for the dielectric, respectively, and *ɛ_s_* and *d* are the permittivity and thickness of the semiconductor. The inverse of the derivative of the interface potential w.r.t. channel potential is used in calculating the hole density as a function of interface potential:

(4) $$p\;\left(V_{0}\right)=\frac{\varepsilon_{0}\varepsilon_{i}^{2}}{\varepsilon_{s}l^{2}e}\;U_{GS}{\left(\frac{dV_{0}}{dU_{GS}}\right)}^{-1}$$

Finally, the derivative of the hole population with respect to the interface potential gives the number density of traps at an energy *E* above the valence band (i.e., the trap‐DOS):

(5) $$N\left(E\right)\approx \frac{1}{e}\frac{dp\left(V_{0}\right)}{dV_{0}}$$

After extracting representative trap‐DOS spectra from experimental samples, the following double exponential was fit to a typical device:

(6) $$N\left(E\right)=N_{1}\exp\left(-\frac{E}{E_{1}}\right)+N_{2}\left(-\frac{E}{E_{2}}\right)$$

with *E_1_* = 24.4 meV and *E_2_* = 71.2 meV as characteristic decay energies, and *N_1_* = 7.0 × 10^19^ eV^−1^ cm^−3^ and *N_2_* = 3.5 × 10^17^ eV^−1^ cm^−3^ are the amplitudes of the respective exponential distributions. The Atlas software samples this function at 48 points evenly spaced from *E* = 0 to 1.7 eV (i.e., the breadth of the bandgap) with linear interpolation.

The simulated drain‐source voltage was set to −3 V and the gate‐source voltage was varied from 0 to −60 V. Linear mobility was calculated from these simulated transfer curves using the equation:

(7) $$\mu_{lin}=\frac{L}{CWV_{DS}}\;\frac{\partial I_{D}}{\partial V_{GS}}$$

Computations were performed on the Wake Forest University DEAC Cluster, a centrally managed resource with support provided in part by the University.

### Transistor Fabrication

The substrates were cleaned by rinsing with acetone for 30 s; 10 min acetone bath; acetone and isopropyl alcohol (IPA) rinses, 10 s each; 10 min IPA bath; IPA rinse (10 s); N_2_ compressed gas drying; 10 min UV‐Ozone; deionized water rinse for 30 s; N_2_ compressed gas drying. A 3 nm Ti adhesion layer was first deposited at a rate of 1 Å s^−1^ on the substrates through a shadowmask using a Kurt Lesker (KJLC) Spectros electron‐beam deposition system at ≈10^−7^ Torr. Au contacts were then thermally deposited at a rate of 0.5 Å s^−1^ and a thickness of 40 nm. Channel lengths ranged from 30 to 100 µm. Contacts were cleaned following the procedure described for the substrates, and then the contacts were either left plain or treated with a self‐assembled monolayer of PFBT to create contacts with work functions of 5.0 and 5.4 eV, respectively, corresponding to injection barriers of *Φ* = 0.4 eV and *Φ* = 0.0 eV. These *Φ* values were calculated as the difference between the measured work function of the contacts and the 5.4 eV HOMO level of IDT‐BT. While the values were calculated from work function and HOMO level measurements, it was noted that effects like Fermi level pinning and chemical/morphological changes at the contact/semiconductor interface made these values only estimates. Semiconductor films of IDT‐BT were spin‐coated at 208 rad s^−1^ (2000 rpm) for 60 s with a 5 s ramp and then annealed on a hot plate at 100 °C for 10 min. A layer of Cytop fluoropolymer dielectric (CTL‐809‐M) was then spin‐coated at 208 rad s^−1^ (2000 rpm) for 60 s with a 2 s ramp. Cytop solutions were used either undiluted or diluted with CT‐Solv. 180 in ratios up to 1:1 by volume. The Cytop films were cured by placing substrates in a vacuum oven at 50 °C for 8.5 h (including warm‐up time, ≈20 min). Au gate electrodes were thermally deposited at a rate of 2 Å s^−1^ and thickness of 40 nm to complete the top gate, bottom contact devices. Dielectric capacitance was measured by fabricating MIS structures: Cytop solutions were deposited on silicon substrates terminated with a 200 nm SiO_2_ layer using the same spin recipe and 40 nm Au top contacts were thermally deposited through a shadowmask. Quasi‐static capacitance measurements using an HP 4155C semiconductor parameter analyzer directly measured the series capacitance of the SiO_2_/Cytop structure, from which the Cytop capacitance was calculated by solving the circuit equation for series capacitors using the measured (total) capacitance and the known capacitance of the 200 nm SiO_2_ layer (the SiO_2_ layer was used to limit leakage current, thus resulting in a more precise capacitance measurement). Average capacitance was calculated from a minimum of three separate samples and uncertainty was reported as standard deviation.

Source and drain bottom electrodes made of PEDOT: PSS (Clevios PJ700 formulation, purchased from Heraeus) were inkjet printed on a 125 µm thick poly(ethylene 2,6‐naphthalate) (PEN) substrate (purchased from Du Pont) with Fujifilm Dimatix DMP2831 at 40 °C. Channel lengths ranged from 40 to 200 µm. The substrates were pre‐treated before printing by oxygen plasma (80 W for 10 s). The IDT‐BT semiconductor layer and the Cytop dielectric layer were each spin cast and annealed as described above. Finally, a sprayed graphite gate (Bonderite LG‐P, aka “Aerodag G,” purchased through Ted Pella) was applied through a shadow mask to complete the fully‐solution processed transistors. Samples that were used to compare the effects source/drain materials (i.e., Figure 6) used an Au gate thermally deposited through a shadowmask (40 nm 2 Å s^−1^), such that all samples compared had the same semiconductor, dielectric, and gate materials. The PET film remained the substrate for testing the PEDOT: PSS contacts to preserve the printed characteristics.

OFETs with sprayed graphite contacts (Bonderite LG‐P) used the top contact, bottom‐gate staggered structure, which was compatible with the surface roughness of the graphite layer. Highly doped Si^++^ substrates with a 200 nm layer of SiO_2_ were cleaned following the same procedure as above. Cytop was then deposited on top, followed by parylene‐C. Parylene deposition was carried out in a quartz‐tube bespoke reactor under vacuum (≈1 mTorr). Three temperature zones were used: first, the dimer di‐*para*‐xylene (Acros Organics) was heated to 120 °C, at which temperature it sublimes. The material then passed through a 700 °C furnace which splits the dimer into monomers. The monomers then polymerized on the sample surfaces at room temperature to provide a conformal coating. 40 nm Au contacts were deposited using thermal vapor deposition as described above. Dielectric capacitance measurements were then taken following the same procedure described above, using the Au coplanar contacts as the top electrode of the capacitor structure. To finish the devices, first, a semiconductor layer of IDT‐BT was spin‐coat in the same manner as before, and then the graphite spray was used through a shadow mask to form the top source/drain contacts.

### Transistor Characterization

Transfer (*I_D_* vs *V_GS_*) and output (*I_D_* vs *V_DS_*) characteristics were measured with an HP 4155C semiconductor parameter analyzer in ambient atmospheric conditions and in the dark. Gate leakage current was negligible for all devices characterized. Mobility in the linear regime was calculated using Equation (7), where the slope extracted from the transfer curve was used to calculate the value of the partial derivative of *I_D_* w.r.t. *V_GS_*. Mobility in the linear regime was calculated from Equation (7), and mobility in the saturation regime was calculated using the equation:

(8) $$\mu_{sat}=\frac{2L}{WC}\;\;{\left(\frac{\partial \sqrt{I_{D}}}{\partial V_{GS}}\right)}^{2}$$

where the partial derivative was extracted from a best‐fit line of the square root of *I_D_* versus *V_GS_*. In the case of non‐ideal *I_D_* versus *V_GS_* curves (e.g., shifted threshold voltage), the best‐fit line was applied to the region suggested by Choi, et al. to avoid mobility overestimation.^[^ ^54^ ^]^ Uncertainty was reported as a standard deviation. Bias conditions for linear mobility measurements were *V_DS_* = −3 to −5 V; *V_DS_* values for the saturation regime were adjusted such that the device stayed in the saturation regime as determined by *V_DS_* > *V_GS_* and verified by the output characteristics. Typical values for saturation *V_DS_* ranged from −40 to −80 V. *V_GS_* was taken out to a minimum of −20 V but up to −80 V, as limited by electrical shorting through the gate, which occurs at lower *V_GS_* vales for high‐*C* devices because of the corresponding higher field strength.

Contact and channel resistance were calculated using the gated‐transfer length method (g‐TLM). The g‐TLM model assumes that the total device resistance (*R_tot_*) is the sum of two components, the contact resistance (*R_C_*, which includes both the source and drain contact resistances) and the channel resistance (*R_Ch_*). The channel resistance is assumed to be directly proportional to the channel length, and so when the total resistance is plotted as a function of length, the *y‐*intercept yields the contact resistance while the slope is the channel resistance (per unit length). According to the model, the channel resistance i−1s directly related to the channel mobility (*μ_ch_*), that is, the mobility in the absence of contact effects, through

(9) $$\mu_{Ch}=\frac{L}{R_{ch}WC\left(V_{GS}\;-\;V_{th}\right)}$$

### Statistical Analysis

Aggregate data were expressed as mean ± standard deviation. Analysis and plotting were carried out using Origin Pro.

## Conflict of Interest

The authors declare no conflict of interest.

## Supporting information



Supporting Information

## Data Availability

The data that support the findings of this study are available from the corresponding author upon reasonable request.
