# Supplementary material for: Designing Contact Independent High‐Performance Low‐Cost Flexible Electronics
Source: Adv Mater. 2024 Oct 9;36(48):2410442. doi: 10.1002/adma.202410442 (PMC11602682; doi:10.1002/adma.202410442)
Supplement: Supplementary file 1 — Supporting Information [file ADMA-36-2410442-s001.pdf]

ADVANCED MATERIALS

Supporting Information

for *Adv. Mater.*, DOI 10.1002/adma.202410442

Designing Contact Independent High-Performance Low-Cost Flexible Electronics

*Matthew Waldrip, Yue Yu, Derek Dremann, Tommaso Losi, Benjamin Willner, Mario Caironi, Iain McCulloch and Oana D. Jurchescu**

Supporting Information

Designing contact independent high-performance low-cost flexible electronics

*Matthew Waldrip, Yue Yu, Derek Dremann, Tommaso Losi, Benjamin Willner, Mario Caironi, Iain McCulloch, and Oana D. Jurchescu**

Dr. M. Waldrip, Y. Yue, D. Dremann, Prof. O. D. Jurchescu

Department of Physics and Center for Functional Materials, Wake Forest University, Winston-Salem, NC 27109 USA

E-mail: jurchescu@wfu.edu

Dr. T. Losi, Prof. M. Caironi

Center for Nano Science and Technology, Istituto Italiano di Tecnologia, Via Rubattino 81, 20134 Milano, Italy

Dr. B. Willner, Prof. I. McCulloch

Department of Chemistry, Chemistry Research Laboratory, University of Oxford, Oxford, OX1 3TA, UK

Prof. I. McCulloch

Andlinger Center for Energy and the Environment, and Department of Electrical and Computer Engineering, Princeton University, Princeton, NJ, 08544, USA

*

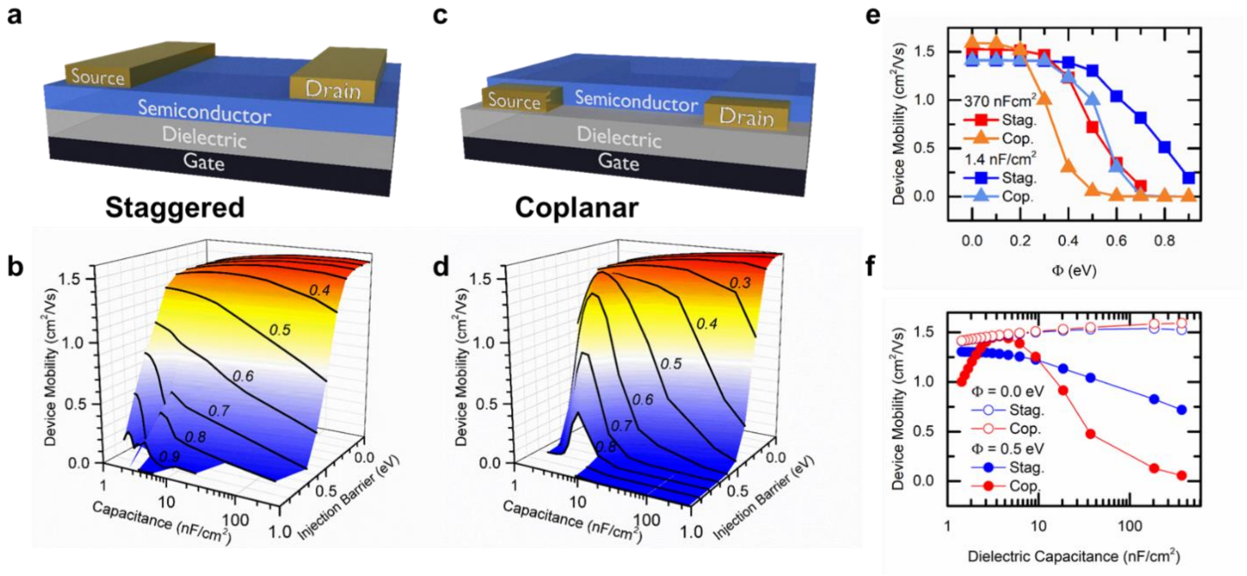

Figure S1. Simulation results for intrinsic semiconductor mobility set to 1.6 cm^2/Vs. a) Simulated bottom-gate, top-contact staggered structure. b) Corresponding device mobility vs dielectric capacitance and injection barrier. Black lines are curves at a constant injection barrier. c) Simulated bottom-gate, bottom-contact coplanar structure. d) Corresponding device mobility vs dielectric capacitance and injection barrier. e) Device mobility as a function of injection barrier for high-capacitance devices (staggered, red squares; coplanar, orange triangles) and low-capacitance devices (staggered, blue squares; coplanar, light blue triangles). f) Device mobility as a function of dielectric capacitance for Φ = 0 eV (open circles) and Φ = 0.5 eV (filled circles).

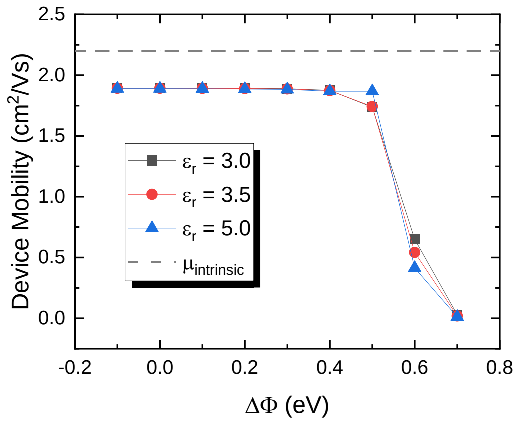

Figure S2. Device mobility as a function of injection barrier with μ_i = 2.2 cm^2/Vs with different values of semiconductor dielectric constant.

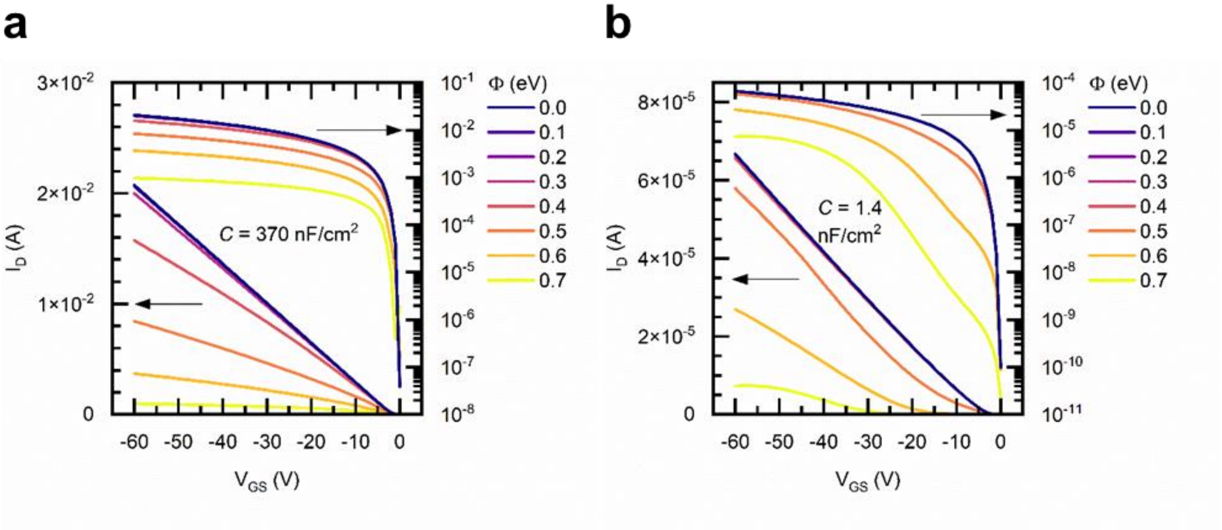

Figure S3. Sample transfer curves from TCAD simulations for staggered devices. a) I_D vs V_{GS} curves for a high-capacitance OFET (C = 372 nF/cm^2) with different injection barriers. V_{DS} = -3 V for all curves. b) I_D vs V_{GS} curves for a low-capacitance OFET (C = 1.4 nF/cm^2) for different values of the injection barriers. V_{DS} = -3 V for all curves.

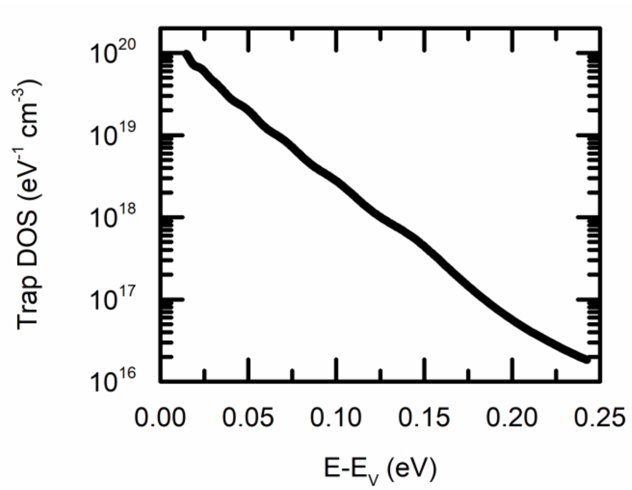

Figure S4. Trap density of states spectrum extracted from an experimental OFET. The t-DOS spectrum for a typical IDT-BT OFET with Cytop dielectric as extracted via the Grunewald method, which follows a characteristic double-exponential. A fit to such a spectrum was programmed into the TCAD simulations. Small oscillations in the curve are attributed to polynomial fitting in the Grünewald solution.

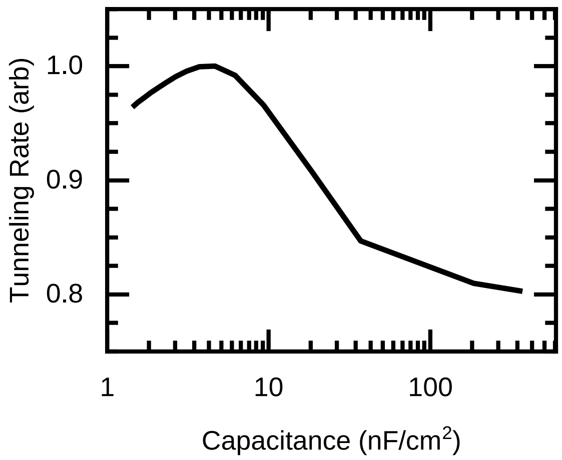

Figure S5. Tunneling rate near the injection contact of simulated coplanar FETs with a 0.5 eV injection barrier (normalized to value at 3.7 nF/cm^2). The tunneing rate is calculated by the software at each grid point in the semiconductor during the simulation, and the data here were selected at a point in the channel near the source contact.

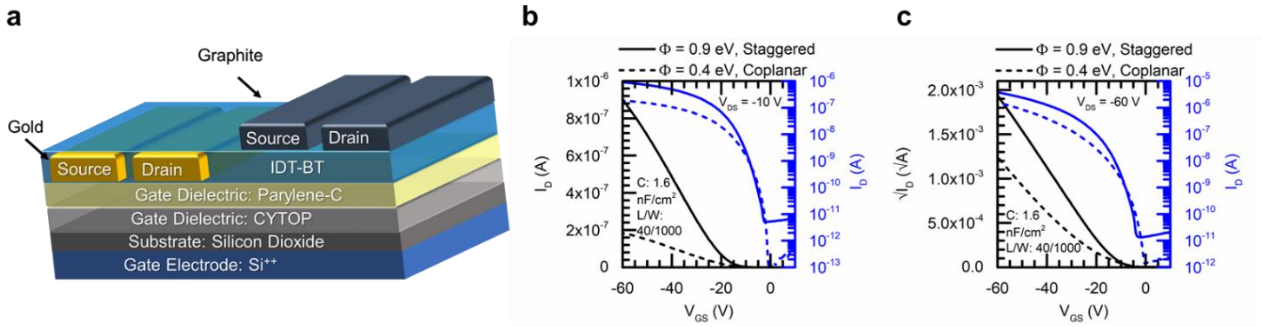

Figure S6. Comparison of OFET performance in staggered vs coplanar geometry with an injection barrier. a) Structure used to test coplanar Au contacts (Φ = 0.4 eV) and staggered graphite-spray contacts (Φ = 0.9 eV) on the IDT-BT film. The vapor-deposited layer of parylene-C facilitated the spin-coating of the polymer semiconductor, since the Cytop layer is extremely hydrophobic. The capacitance of the triple-layer dielectric was directly measured to be C = 1.6 nF/cm^2. b) Linear transfer curves comparing the staggered, Φ = 0.9 eV contact (solid lines) to the coplanar, Φ = 0.4 eV contact (dashed lines). Despite the injection barrier of the staggered contact being twice as large as that of the coplanar contact, the staggered structure devices had an average linear mobility 3.5 times as large as the coplanar structure, μ_{lin} = 0.07 ± 0.01 cm^2/Vs *vs* μ_{lin} = 0.02 ± 0.01 cm^2/Vs, respectively. c) Saturation transfer curves comparing the staggered, Φ = 0.9 eV contact (solid lines) to the coplanar, Φ = 0.4 eV contact (dashed lines). The staggered structure devices averaged μ_{sat} = 0.12 ± 0.03 cm^2/Vs while the coplanar structure devices averaged μ_{sat} = 0.06 ± 0.02 cm^2/Vs, a factor of two difference.

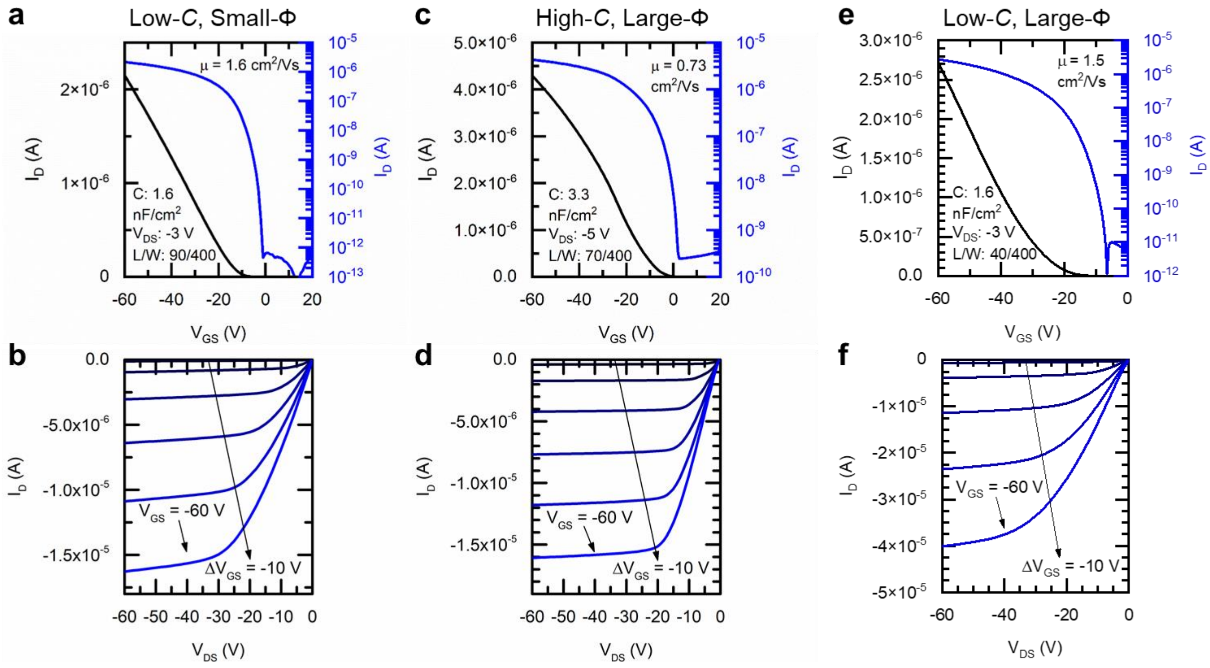

Figure S7. Example transfer and output characteristics for devices of different C and Φ. a) Transfer and (b) output curves for the low-*C* (1.6 nF/cm^2), Φ = 0 eV device. c) Transfer curve of the high-*C* (3.3 nF/cm^2), Φ = 0.4 eV device and d) corresponding output curve. e) Transfer and f) output characteristics for a device with low-*C* (1.6 nF/cm^2), and Φ = 0.4 eV.

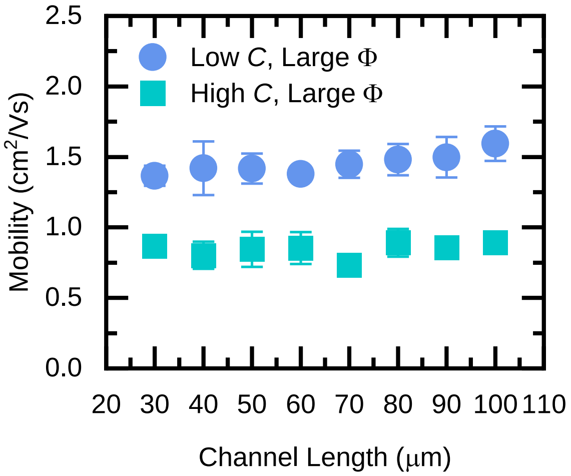

Figure S8. Device mobility with respect to channel length for devices with Φ = 0.5 eV. The absence of a dependence of mobility on channel length suggests that long channel lengths are not necessary to recover device performance in the presence of a large injection barrier, and thus devices could be further downscaled with minimal impact at this low capacitance.

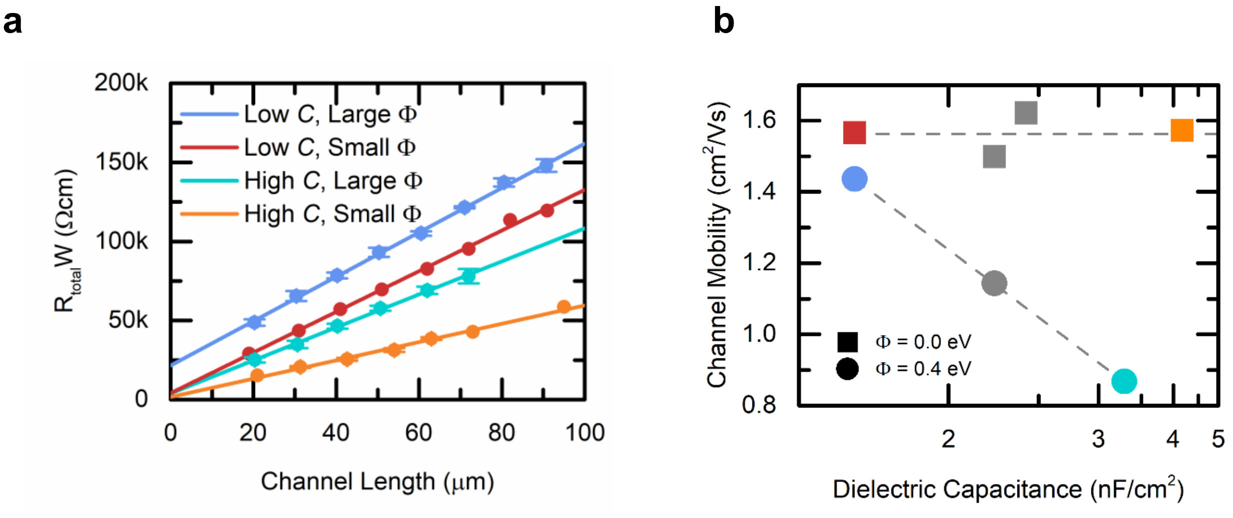

Figure S9. Gated-transfer length method (gTLM) analysis of experimental OFETs. a) Width-normalized total resistance plotted as a function of channel length, corresponding to the data sets in Fig. 3. Lines are linear regressions to data. The y-intercept gives the width-normalized contact resistance, while the slope is equivalent to the width-normalized channel resistance per micrometer. Drain-source voltage for the Low *C*, Large *Φ*; Low *C*, Small *Φ*; and High *C*, Small *Φ* samples was V_{DS} = -3 V. Drain-source voltage for the High *C*, Large *Φ* samples was V_{DS} = -5 V. b) Channel mobility, calculated from gTLM channel resistance, for *Φ* = 0 eV (squares) and *Φ* = 0.4 eV (circles). Colored points correspond to devices in a. Lines are guides for the eye.

Supplementary Table 1. Experimental contact resistance, channel resistance and channel mobility values from gated-TLM analysis; device mobility extracted from I_D vs. V_{GS} curves

	$R_C W$ (kΩcm)	$R_{Ch} W$ (kΩcm)*	μ_{ch} ($cm^2V^{-1}s^{-1}$)	μ_{device} ($cm^2V^{-1}s^{-1}$)
Φ = 0.0 eV, *C* = 1.6 ± 0.1nF/cm²	4.1 ± 1.8	39 ± 1	1.6 ± 0.1	1.7 ± 0.1
Φ = 0.0 eV *C* = 3.7 ± 0.2 nF/cm²	1.7 ± 1.1	17 ± 1	1.6 ± 0.1	1.5 ± 0.2
Φ = 0.4 eV *C* = 1.6 ± 0.1 nF/cm²	21.6 ± 1.4	42 ± 1	1.4 ± 0.1	1.5 ± 0.1
Φ = 0.4 eV *C* = 3.7 ± 0.2 nF/cm²	3.8 ± 1.6	31 ± 1	0.87 ± 0.03	0.8 ± 0.1

*R_{Ch} normalized to *W* = 30 μm channel length

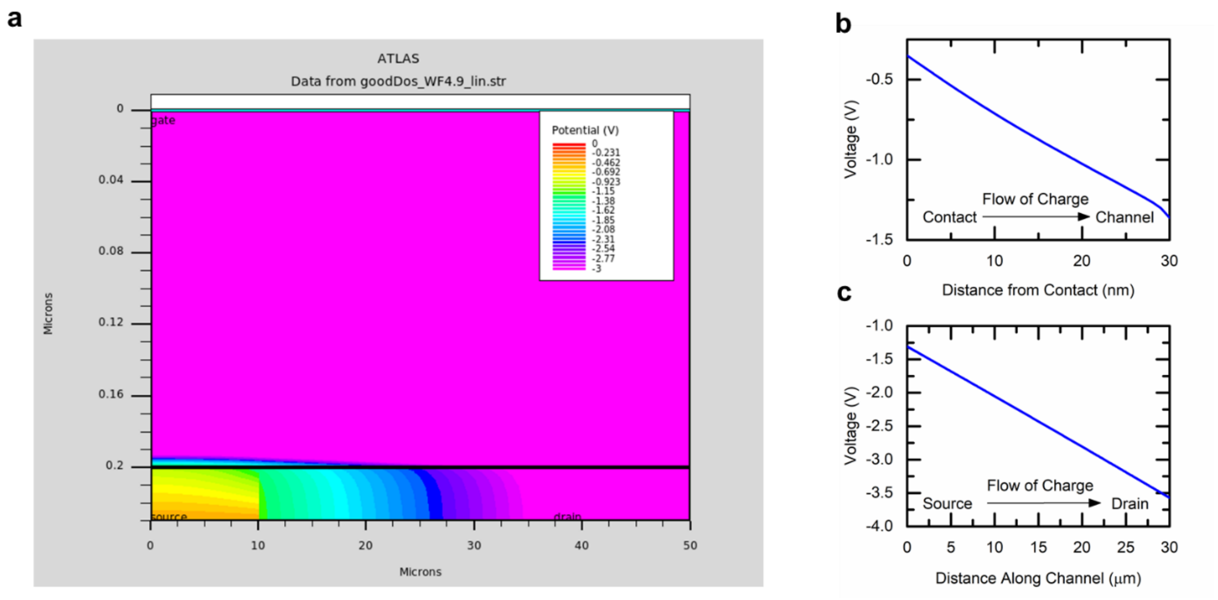

Figure S10. Potential map and cut-lines calculated through TCAD simulation. a) Two-dimensional FEA map of potential in a simulated staggered device (top gate, bottom contact) under the bias conditions of V_{GS} = -60 V . Note aspect ratio, i.e., y-dimension scale is much smaller than x-dimension. Example data is for C = 9.3 cm^2/Vs, Φ = 0.5 eV. b) Cut-line of potential going from edge of contact (x = 9.99 μm, y = 0.23 μm) to channel (9.99 μm, 0.2 μm). Coordinates for cut-lines were chosen to not lie on a boundary point in order to avoid ambiguity at the interface. In all simulated devices with Φ = 0.5 eV, the potential drop at the injection interface was -0.351 V (source is 0 V), which corresponds to the interface component of contact resistance, $R_{C,int}$. The voltage drop across the bulk fluctuated with dielectric thickness/capacitance, providing further evidence that the electric field in the bulk is responsible for the observed trends. Here, $V_{S,bulk}$ = 1.009 V for a total contact voltage of V_S = 1.360 V. c) Cut-line of voltage potential across the channel, beginning at (10, 0.201) and ending at (40, 0.201), i.e., the distance of the channel between the source and drain contacts. In the linear regime ($V_{DS} \ll V_{GS}$), a linear potential drop across the channel is expected; however, because of the voltage drop from the contact to the channel, the channel voltage is reduced from the applied V_{DS}. Here, the channel voltage is 2.26 V.

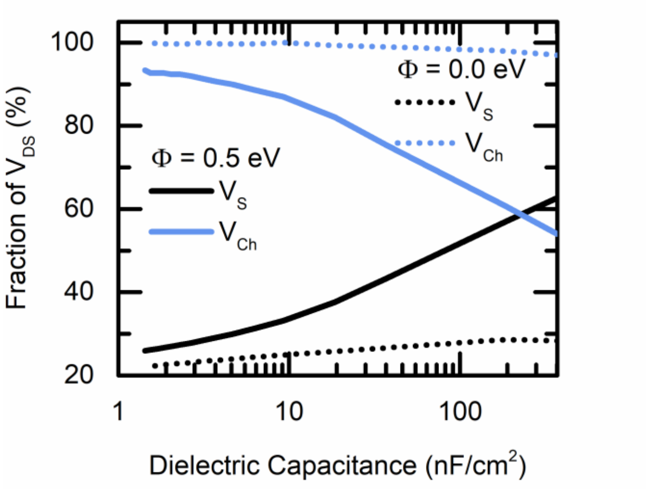

Figure S11. Simulated contact and channel voltage as a function of dielectric capacitance for an intrinsic semiconductor mobility of 1.6 cm^2/Vs. When Φ = 0 eV (dotted lines), the voltage from the source contact to the channel (V_S) increases only by 6.4% of V_{DS} throughout the range of dielectric capacitance range, and correspondingly the channel voltage (V_{Ch}) drops only by 3.0%. However, when Φ = 0.5 eV, V_S increases sharply with increasing C, especially at values of C > 10 nF/cm^2, up to a 37.7% fraction of V_{DS} increase for the range shown here. Because of this voltage drop from the injecting source contact to the channel, V_S is reduced from 93.3% to 54.0% of V_{DS}. The crossover point where V_S becomes greater than V_S is predicted to be approximately C = 230 nF/cm^2, which is a higher value than what is predicted for when the intrinsic mobility is set to 10 cm^2/Vs at C = 40 nF/cm^2. One explanation is that when decreasing the intrinsic semiconductor mobility, the increase in bulk resistance is proportionally greater than the increase in channel resistance (a reasonable assumption since the intrinsic channel resistance is directly dependent on the semiconductor/dielectric interface, which is unchanged), then there will be a resulting build-up of charge along the injection path to the channel which screens the injection field.

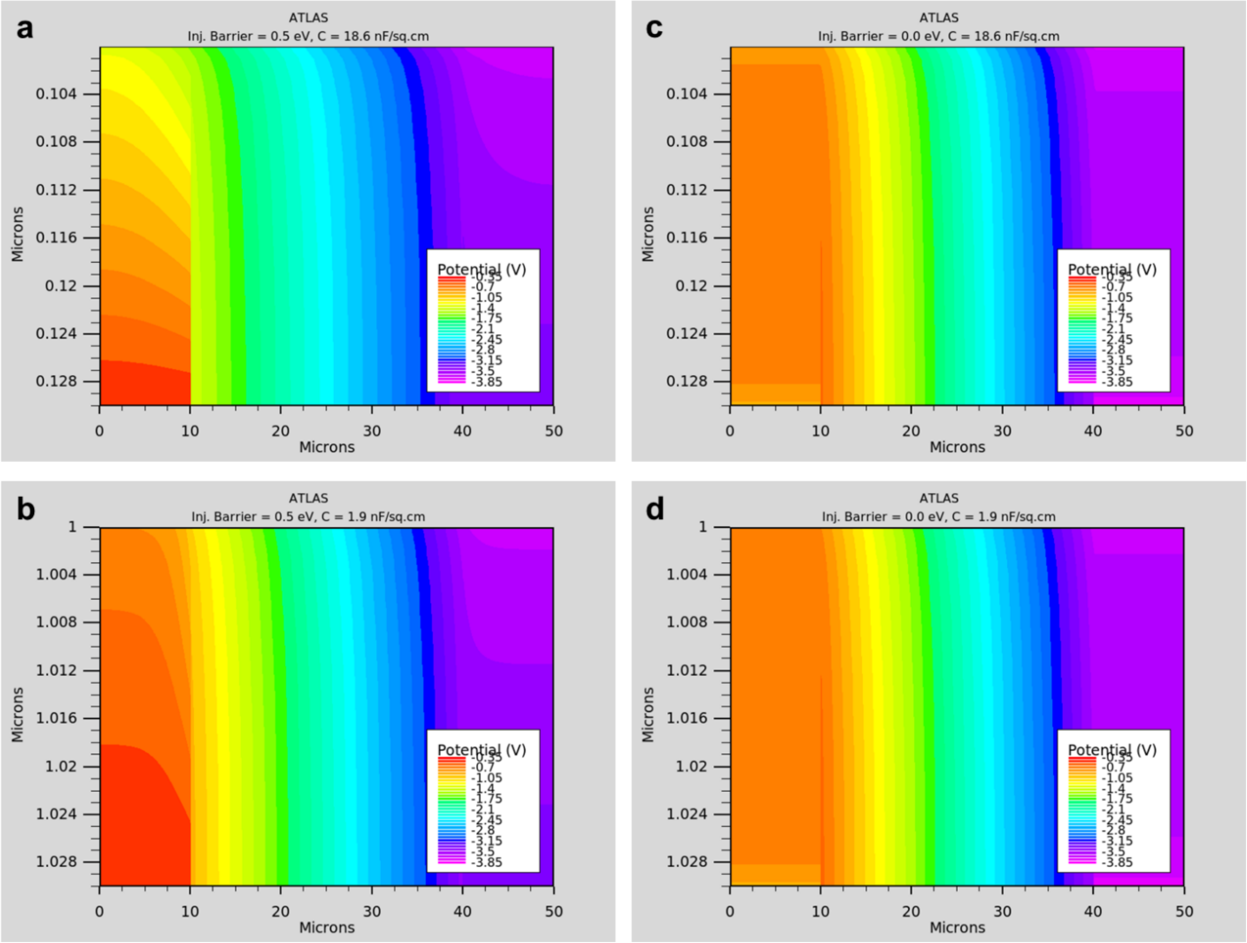

Figure S12. 2D map of the potential distribution in the semiconductor layer (simulation results) In each panel the contacts are located at the bottom with zero thickness (simulating a top-contact device that is upside-down), with the source spanning the range x = (0 μm, 10 μm) and the drain x = (40 μm, 50 μm). In between source and drain is the channel, x = (10 μm, 40 μm); the top boundary of the plot is the semiconductor/dielectric interface. In each of these simulations, the bias conditions are V_{DS} = -3 V (source is set to 0 V) and V_{GS} = -60 V. a. High-capacitance device with Schottky contacts. b. High-capacitance device with ideal contacts. c. Low-capacitance device with Schottky contacts. d. Low-capacitance device with ideal contacts.

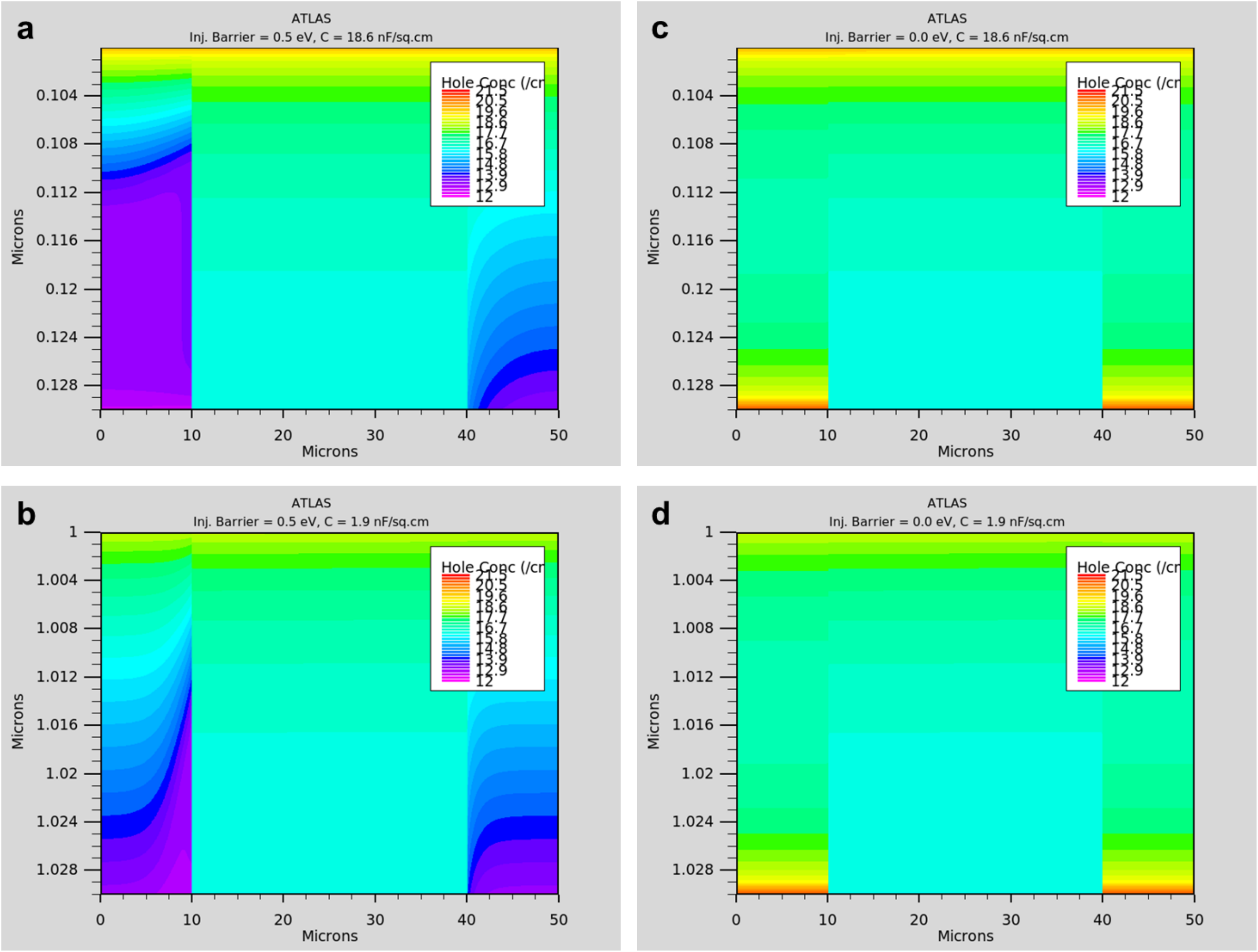

Figure S13. 2D map of charge density in the semiconductor layer (simulation results) See Figure S11 for description of structure and bias conditions. a. High-capacitance device with Schottky contacts. Charge injection is limited by the Schottky barrier, leading to depleted regions in the bulk. b. High-capacitance device with ideal contacts. Note the high charge density near the contact edge which screens the gate field. c. Low-capacitance device with Schottky contacts. d. Low-capacitance device with ideal contacts, with a high charge density near the contact edges.

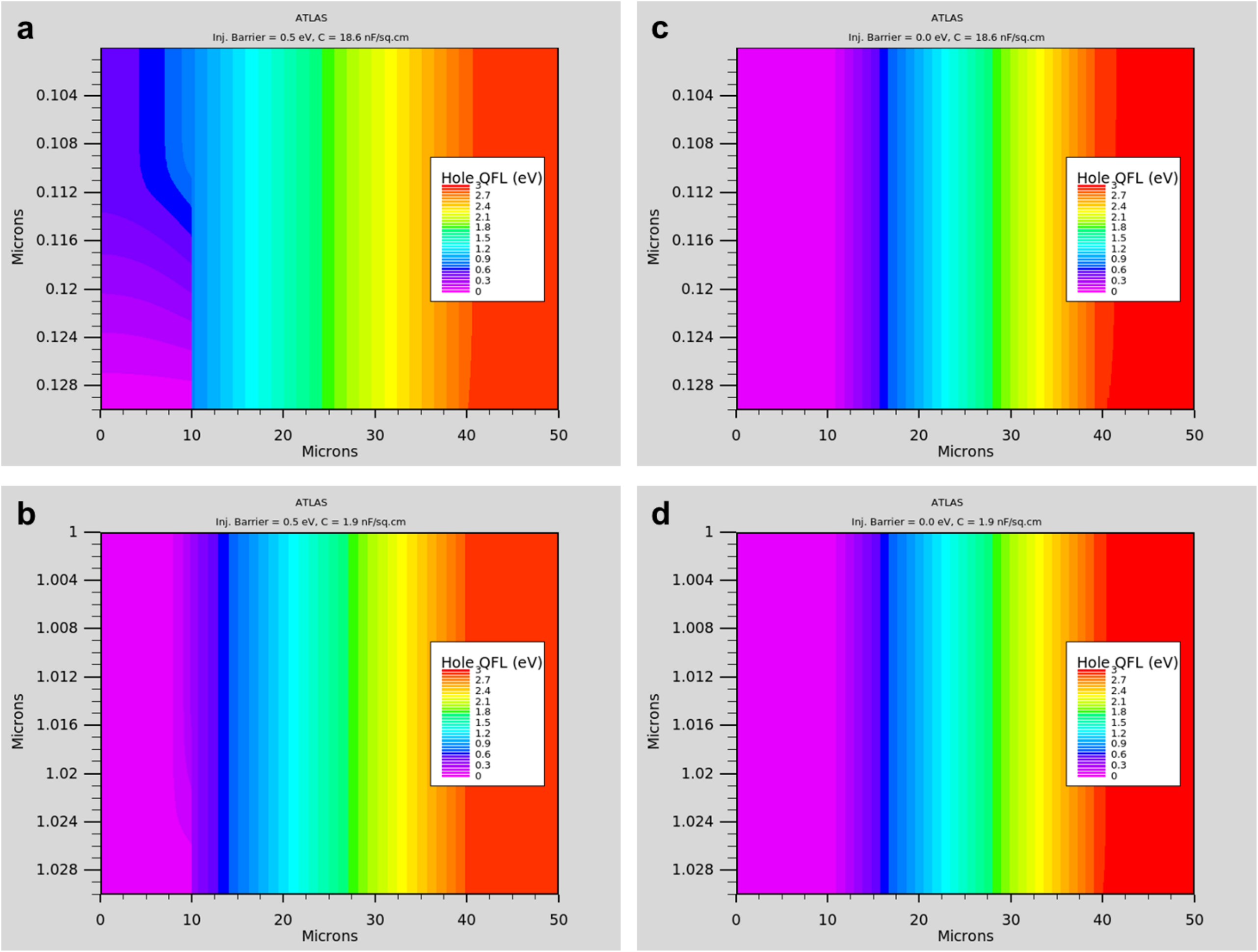

Figure S14. 2D map of the hole quasi- Fermi level (QFL) (simulation results). See Figure S11 for description of structure and bias conditions. a. High-capacitance device with Schottky contacts. b. High-capacitance device with ideal contacts. c. Low-capacitance device with Schottky contacts. d. Low-capacitance device with ideal contacts.

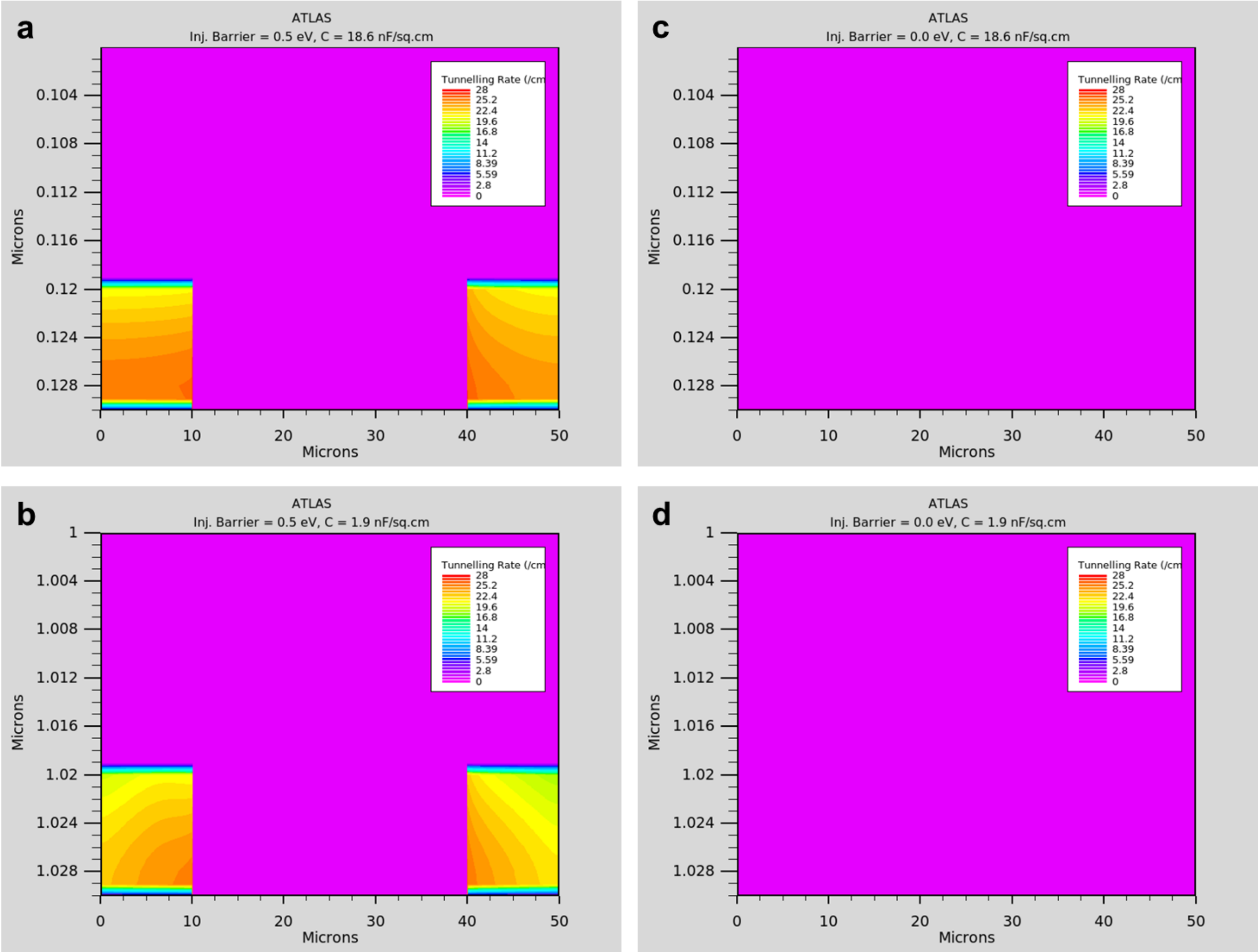

Figure S15. 2D map of the tunneling rate in OFETs (simulation results). See Figure S11 for description of structure and bias conditions. a. High-capacitance device with Schottky contacts, which creates a barrier that is tunneled through. b. High-capacitance device with ideal contacts; charges do not tunnel. c. Low-capacitance device with Schottky contacts. d. Low-capacitance device with ideal contacts.

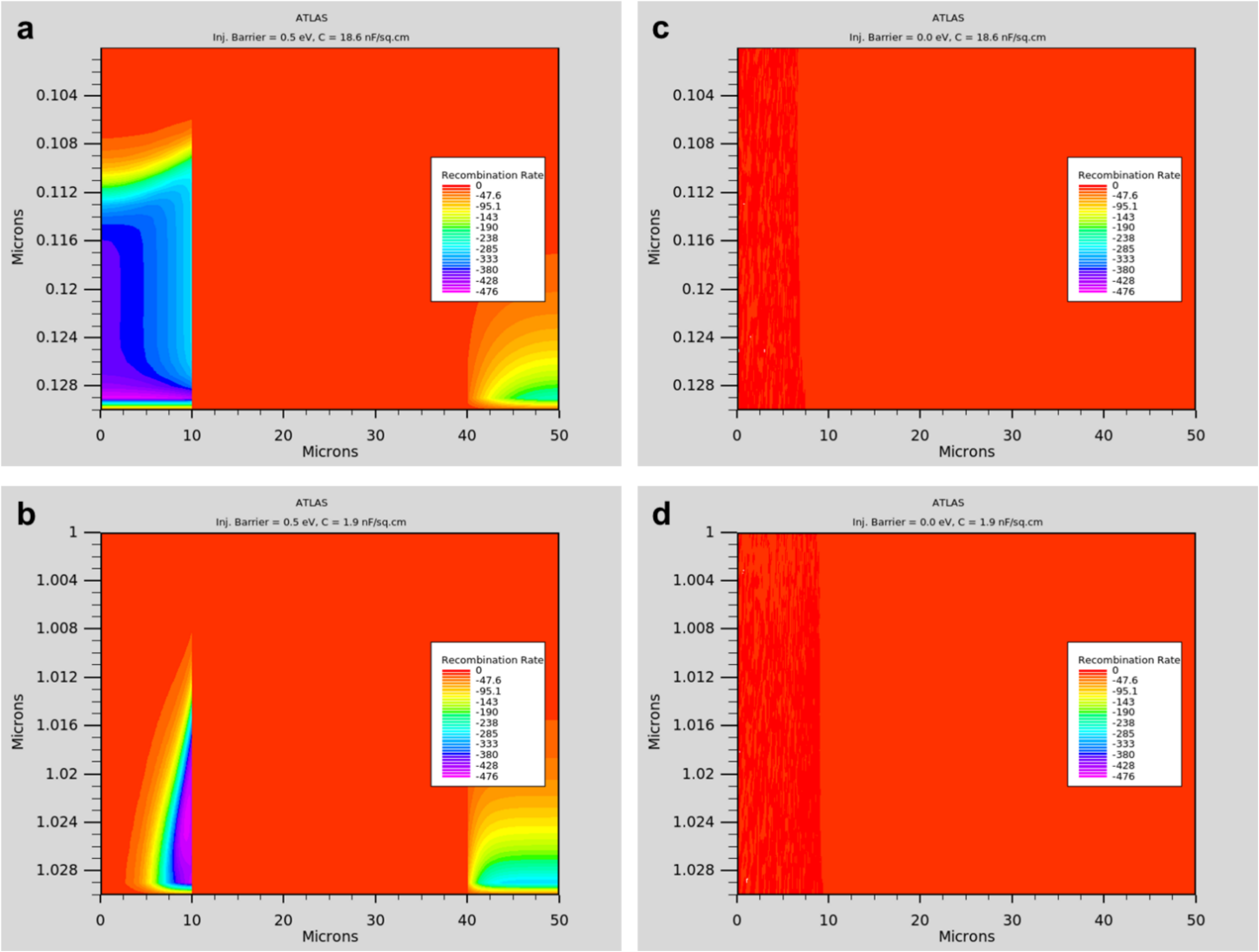

Figure S16. 2D map of the recombination rate in OFETs (simulation results). See Figure S11 for description of structure and bias conditions. In these maps, the more violet color corresponds to greater magnitude of recombination rate (hole recombination is treated as negative by the program). a) High-capacitance device with Schottky contacts. b) High-capacitance device with ideal contacts. c) Low-capacitance device with Schottky contacts. d) Low-capacitance device with ideal contacts.

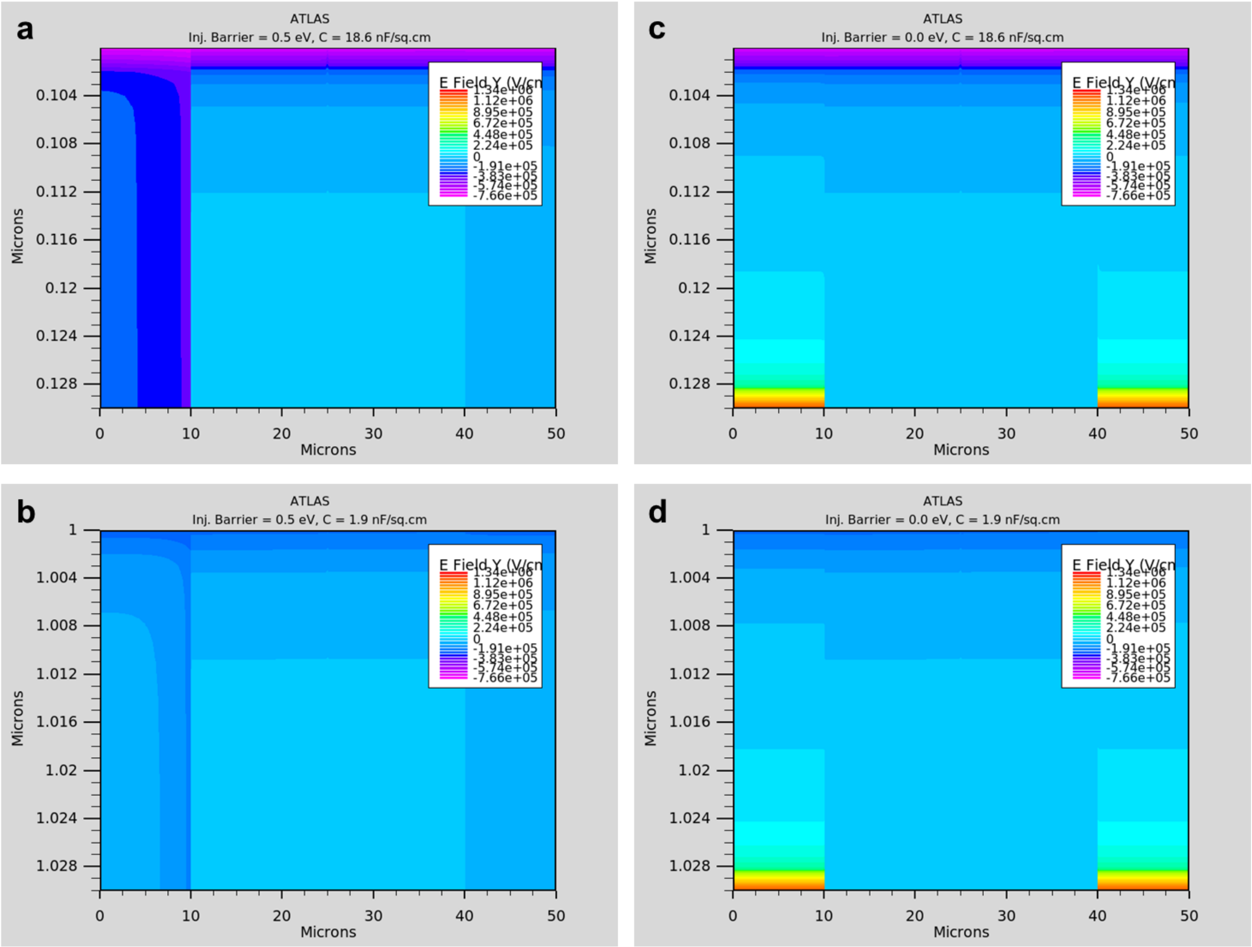

Figure S17. 2D map of E-field Y (E_y) in OFETs (simulation results). See Figure S11 for description of structure and bias conditions. In these maps, the negative values of E_y represent the magnitude of a vector going towards the channel (top), while a positive value represents the magnitude of a vector pointing down. a) High-capacitance device with Schottky contacts. b) High-capacitance device with ideal contacts. Note the electric field near the contacts that is balancing hole injection. c) Low-capacitance device with Schottky contacts. d) Low-capacitance device with ideal contacts.

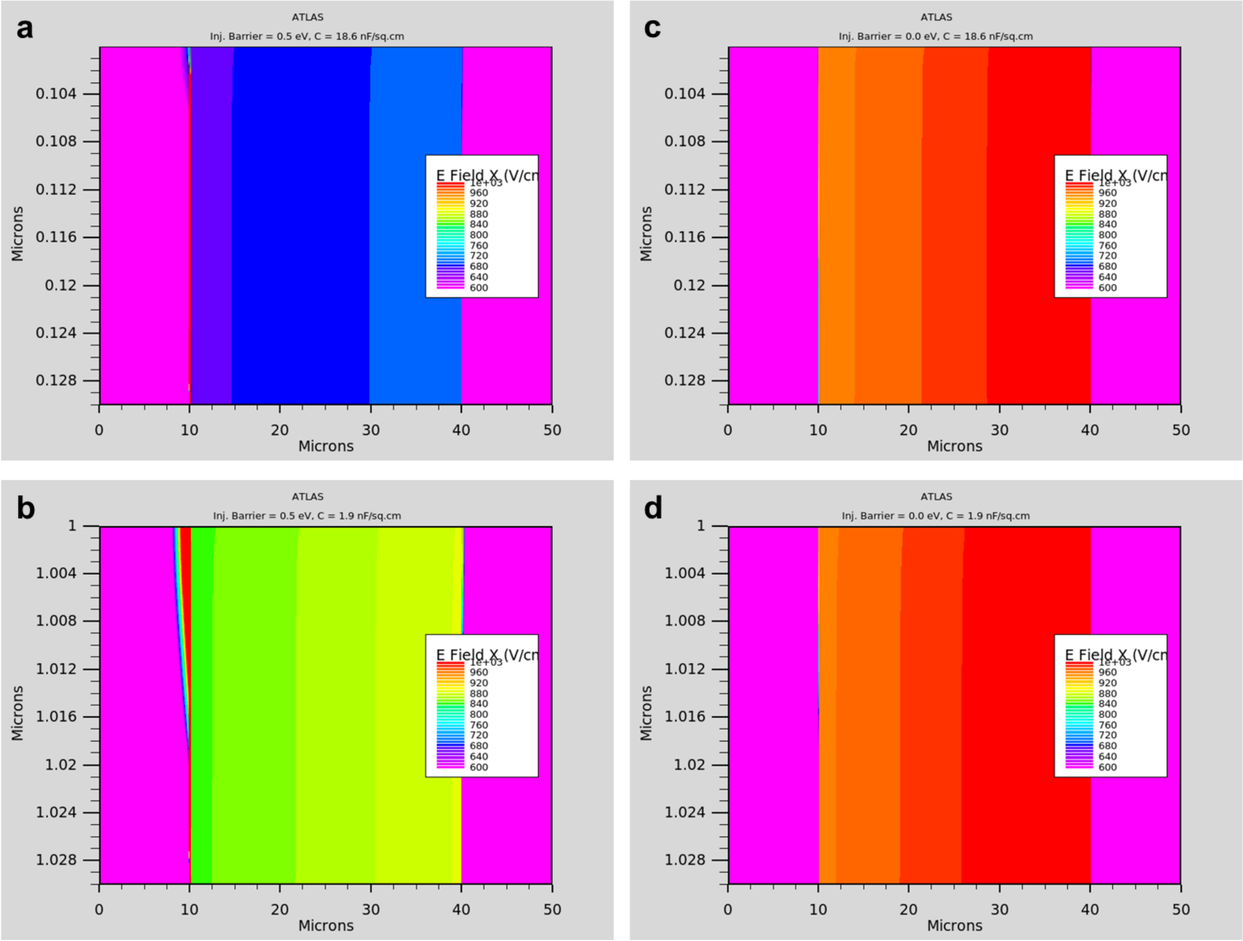

Figure S18. 2D map of E-field X (E_x) in OFETs (simulation results). See Figure S11 for description of structure and bias conditions. a) High-capacitance device with Schottky contacts. Due to the large potential drop from source to channel, this configuration has a much smaller transverse electric field driving the drain current. b) High-capacitance device with ideal contacts. c) Low-capacitance device with Schottky contacts. d) Low-capacitance device with ideal contacts.

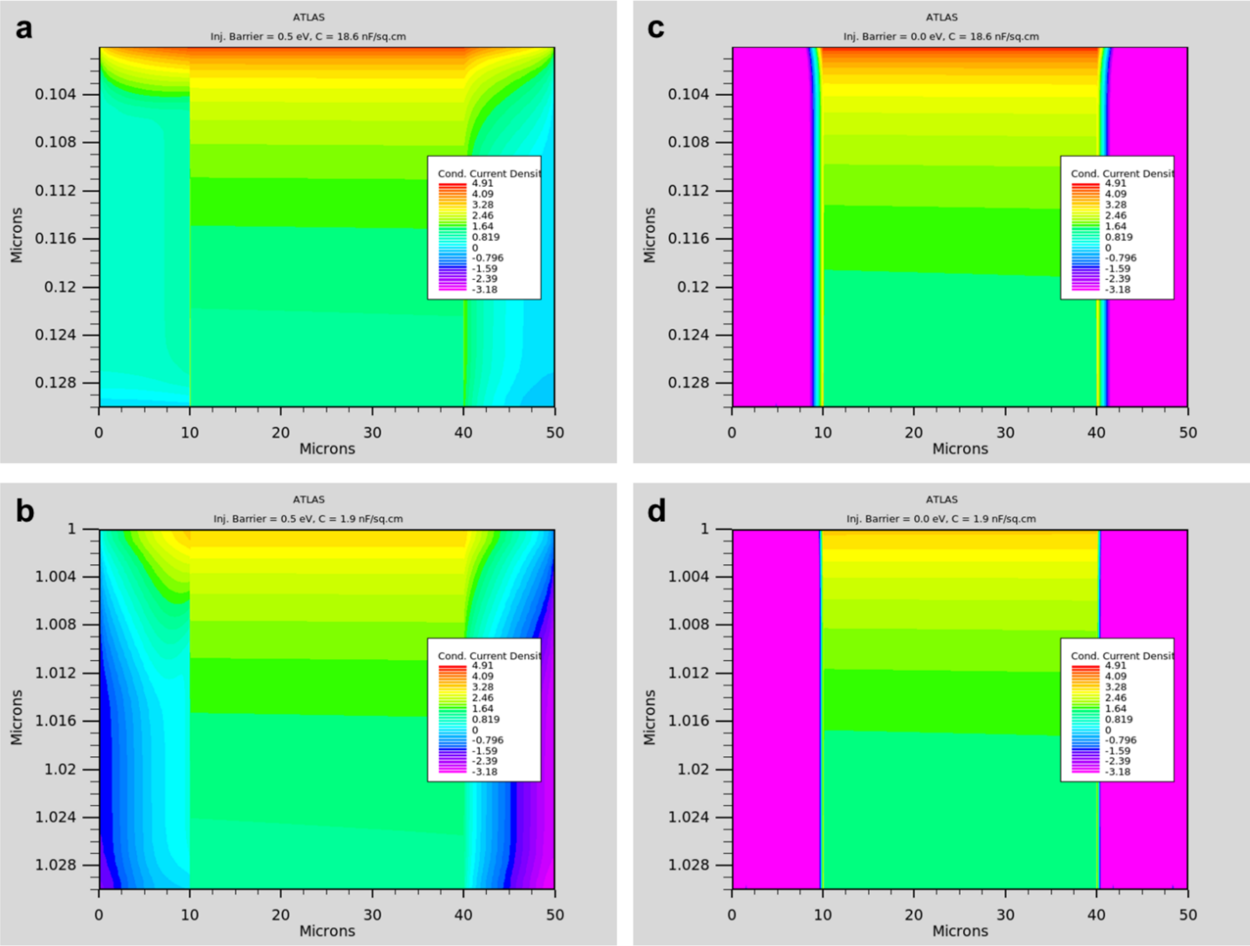

Figure S19. 2D map of current density in OFETs (simulated devices). See Figure S11 for description of structure and bias conditions. Scale corresponds to exponent in log-10 scale. a) High-capacitance device with Schottky contacts. Due to the large potential drop from source to channel, this configuration has a much smaller transverse electric field driving the drain current. b) High-capacitance device with ideal contacts. c) Low-capacitance device with Schottky contacts. d) Low-capacitance device with ideal contacts.

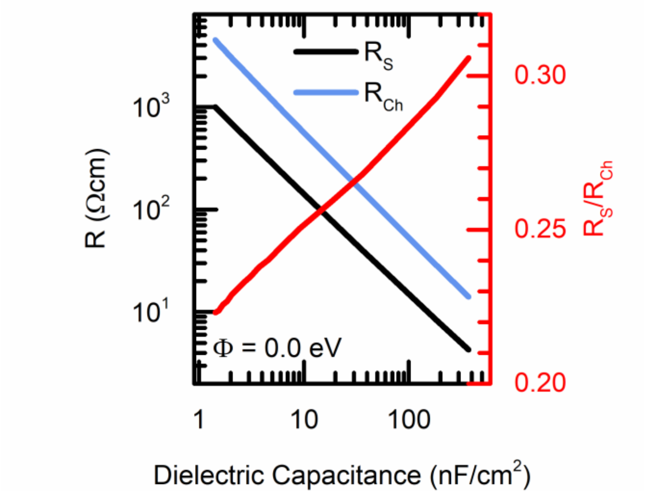

Figure S20. Contact and channel resistance in devices with no injection barrier (simulation results). Source contact resistance (black, left axis), channel resistance (blue, left axis) and the ratio of contact to channel resistance (red, right axis) as a function of dielectric capacitance. Here, the contact resistance only slowly approaches the value of the channel resistance and never surpasses it in the range of dielectric capacitance simulated.

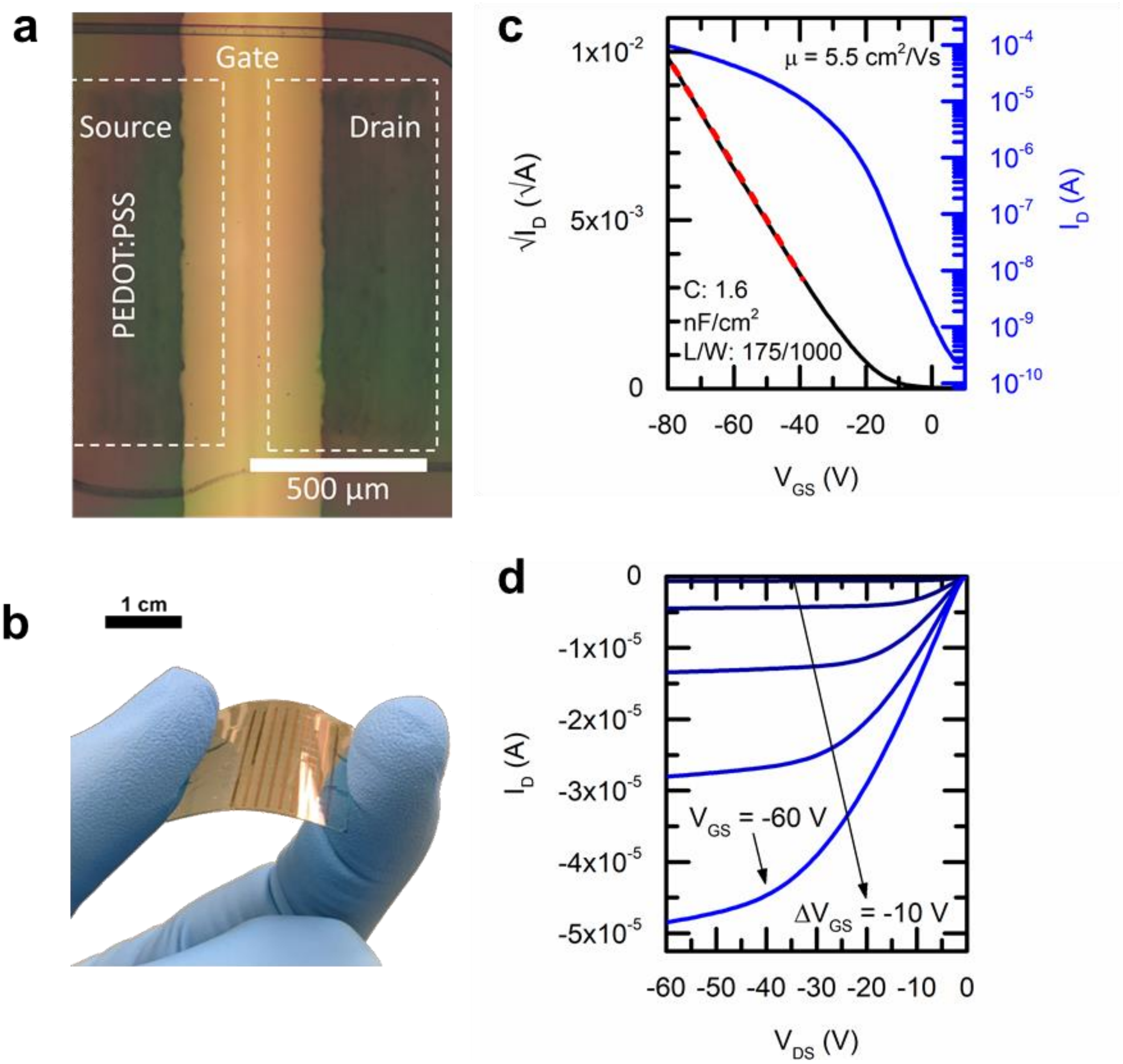

Figure S21. High-performance organic transistors with printed contacts on flexible substrates (Au gate) a) Micrograph of an OFET with printed PEDOT:PSS source/drain contacts, Cytop dielectric and Au gate electrode. Note that the PEDOT:PSS is nearly transparent. b) OFET device arrays on a PET substrate. c) I-V curves in the saturation regime (V_{DS} = 100 V to ensure saturation regime) for the device displaying the highest mobility of the PEDOT:PSS set (Au gate), 5.5 cm^2/Vs. Dotted red line represents the segment of the I-V curve from which mobility was calculated. d) Corresponding output I-V curves of the same device taken from V_{GS} = 0 V to V_{GS} = -60 V.

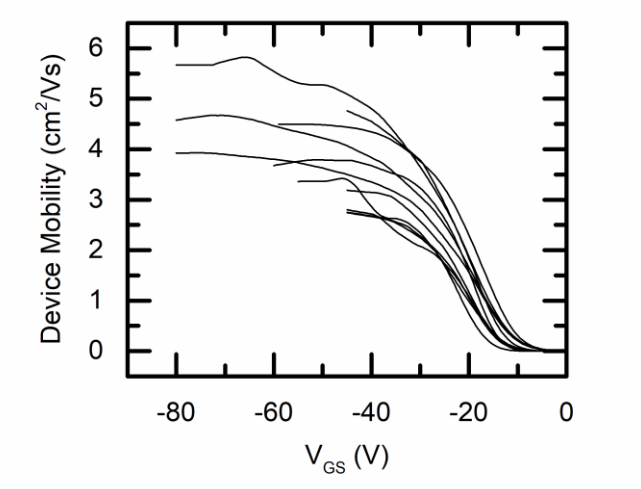

Figure S22. Device mobility vs gate-source voltage for OFETs with printed PEDOT:PSS contacts. The device mobility in the saturation regime for flexible OFETs with printed PEDOT:PSS contacts. V_{DS} ranged from -50 V to -100 V. The curves follow a characteristic up-swing as the device turns on followed by a flattening as the device reaches its on state. Mobility overestimation due to contact resistance/gated contacts would show up as a peak during device turn-on (low V_{GS}), a feature that is absent in these curves.

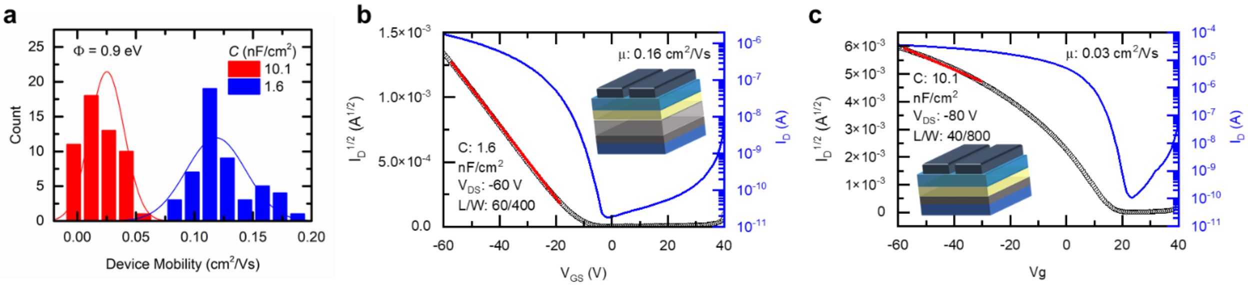

Figure S23. Device mobility vs device capacitance when *Φ* = 0.9 eV a) Histogram comparing device mobility with low and high dielectric capacitance values. OFETs are in the BGTC staggered structure with spray-on graphite contacts with a work function of *Φ* = 0.9 eV, as measured by Kelvin probe. When *C* was decreased from 10.1 nF/cm^2 (red) to 1.6 nF/cm^2 (blue), the mobility increased from 0.02 ± 0.01 cm^2/Vs to 0.12 ± 0.03 cm^2/Vs, an order of magnitude improvement. However, this mobility value is still an order of magnitude lower than the *Φ* = 0.4 eV or *Φ* = 0.0 eV devices. This exemplifies that, while the trend of decreasing *C* to increase device mobility still holds true, performance recovery cannot be achieved for such high values of injection barrier. b) Typical I-V curve in the saturation regime of a *C* = 1.6 nF/cm^2 device, showing good linearity and low turn-on voltage. c) Typical I-V curve in the saturation regime for a *C* = 10.1 nF/cm^2 device. Here, there is a pronounced double-slope in the curve, which signifies that the device performance is severely affected by contact resistance. The turn-on voltage is also notably more positive.

Supplementary Table 2. Experimental contact resistance from gated-TLM analysis for contact types identified in Figure 6 (data-driven devices).

	$R_C W$ (kΩcm)
Graphite	315 ± 140
PEDOT:PSS	22 ± 16
Au	21.6 ± 1.4
Au + PFBT	4.1 ± 1.8

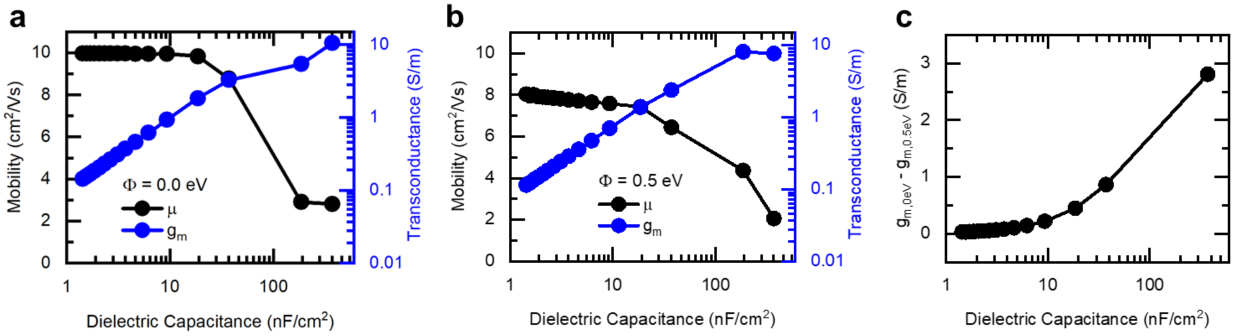

Figure S24. Mobility and transconductance at constant current (simulation results) a) Simulated mobility at constant current (black, left axis) and corresponding transconductance for Φ = 0 eV devices. Here, the steep drop in mobility when C > 100 nF/cm^2 is due to the transistor operating in the subthreshold regime; still the transconductance is high. b) Simulated mobility at constant current (black, left axis) and corresponding transconductance for Φ = 0.5 eV devices. The mobility is still higher at lower values of C when mobility is measured at a constant current value. However, the transconductance increases with C. c) Difference in transconductance between Φ = 0 eV and Φ = 0.5 eV devices. Transconductance varies by only a small amount for smaller values of C (less than 10 nF/cm^2), but a large difference in g_m is present at values of C > 10 nF/cm^2.

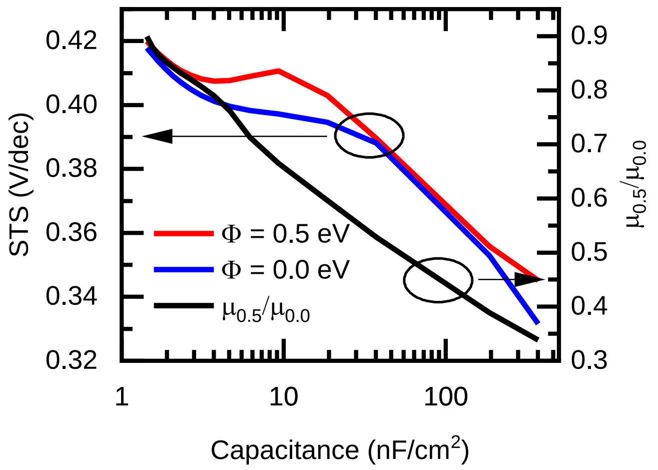

Figure S25. Sub-threshold slope and ratio of mobilities of devices with and without an injection barrier (simulation results). The simulated change in sub-threshold slope varied by only 22% across the entire capacitance range, from 0.42 V/dec to 0.33 V/dec, and the value of the injection barrier has little effect on STS. While it is expected that lowering the capacitance will cause an undesired increase the STS, it is not fundamental that the increase be large. According to

$$STS = \frac{k_B T ln(10)}{q}\left(\frac{N_{it} q^2}{C} + 1\right) \qquad \text{(S1)}$$

where k_B is Boltzmann's constant, T is temperature, q is elementary charge, and N_{it} is the number density of interface traps, a change in C will not have a large effect on STS as long as $N_{it}q^2$ is small compared to C. The changes in STS observed here are small when compared to the effect that changing C has on the device mobility, where the ratio of mobility of Φ = 0.5 eV devices to Φ = 0 eV devices ($\mu_{0.5}/\mu_{0.0}$) drops from 90% to 34%.
